# Reporter-expressing viruses for antiviral drug discovery research

**DOI:** 10.3389/fcimb.2025.1645104

**Published:** 2025-12-17

**Authors:** Dimas Fandi Praditya, Danang Waluyo, Tomoyoshi Nozaki

**Affiliations:** 1 Research Center for Vaccine and Drug, National Research and Innovation Agency Badan Riset dan Inovasi Nasional (BRIN), Bogor, West Java, Indonesia; 2 Graduate School of Medicine, The University of Tokyo, Tokyo, Japan

**Keywords:** reporter-expressing virus, antiviral agent, drug discovery, fluorescence protein, bioluminescence protein

## Abstract

Viruses continue to pose major global health challenges, with recent pandemics underscoring the urgent need for effective antiviral therapeutics. While vaccines have reduced the burden of some viral diseases, many remain difficult to control. Antiviral drug discovery relies on identifying and validating suitable targets through both target-based and phenotype-based screening strategies. Traditional antiviral assays are accurate but labor-intensive and not easily adaptable for high-throughput analysis. Advances in reverse genetics have enabled the development of reporter-expressing recombinant viruses, which allow real-time tracking of viral replication and are increasingly used in high-throughput screening. This review highlights the application of fluorescent and bioluminescent reporter systems in antiviral drug discovery, emphasizing their advantages, limitations, and future prospects.

## 1 Introduction

Viruses have threatened human health since the beginning of history, imposing a massive burden on global health and economies (Li et al., 2007; Galassi et al., 2017). In the past two decades, several major outbreaks have emerged, including SARS in 2003 (Middle East), swine flu in 2009 (Mexico), Ebola in 2013 (West Africa), and the COVID-19 pandemic in 2020, which caused widespread morbidity and mortality. Climate change, increasing human-animal interactions, and global mobility have raised the risk of zoonotic disease transmission and pandemic emergence (Gibb et al., 2020; Chala and Hamde, 2021).

Although vaccines have played a crucial role in eradicating viral diseases such as smallpox, measles, rubella, and polio (Hinman, 1999), many viruses, including HIV and hepatitis C, remain difficult to control. The limited availability and efficacy of vaccines highlight the urgent need for effective antiviral drugs.

A critical early step in antiviral drug discovery is identifying a suitable target. Targets must be both efficacious and safe, commonly referred to as “druggable” (Hughes et al., 2011), meaning they are associated with disease progression but not essential for normal human metabolism. Viral enzymes such as proteases, polymerases, helicases, as well as host factors like receptors or kinases, have been explored as potential targets (Majerová and Konvalinka, 2022).

Drug discovery generally follows two major approaches: target-based and phenotype-based screening. In a target-based screening approach, a viral or host protein essential for replication is selected, and chemical libraries are then screened in biochemical assays to identify inhibitors. This approach is high-throughput, mechanistically informative, and relatively cost-efficient. However, inhibitory activity observed *in vitro* often fails to translate into antiviral efficacy in infected cells or organisms, and fewer than 10% of approved small-molecule antivirals have originated from this strategy (Sadri, 2023).

Phenotype-based screening, by contrast, does not presuppose a molecular target. Instead, it monitors the overall effect of compounds on infected cells, typically by measuring viral replication, cytopathic effect, or other disease-relevant phenotypes. This approach has historically yielded the majority of approved antivirals, including ribavirin, interferons, and remdesivir (Moffat et al., 2017). While more physiologically relevant, it requires more complex readouts and is less amenable to automation without specialized tools.

Traditional phenotypic assays differ substantially in throughput, sensitivity, reproducibility, and cost (**Table 1**). The plaque assay remains the gold standard for quantifying infectious progeny due to its high sensitivity for infectious virus. However, it is time-consuming (2–7 days), labor-intensive, and not well-suited for large-scale screening applications. Its reproducibility depends on strict standardized conditions, as variability may arise from multiple factors such as cell type, assay conditions, and operator-dependent plaque counting. Under rigorous quality control, plaque assays can achieve good reproducibility with reported coefficients of variation below 16% (Denani et al., 2025). Recent efforts in standardization and automation have improved consistency, yet the plaque assay is still best reserved for vaccine evaluation and confirmatory studies rather than high-throughput antiviral screening.

**Table 1:** Comparison of advantages and limitations of commonly used phenotypic antiviral screening system.

Performance characteristics	Plaque-forming assay	Immunoassay	Nucleic acid-based assay	Reporter virus-based assay
Throughput	Low	High	High	Very high
Platform	6–24 well	Up to 384 well	Up to 384 well	Up to 1,536 well
Automation	Limited	Yes	Yes	Yes
Cost	Low	High	High	Moderate
Detection sensitivity	High (for infectious particles)	Moderate	High	Very high (for infection detection)
False positive hit	Low	Moderate	High	Moderate
Signal-to-background ratio	Moderate (visual endpoint)	Moderate	High	High
Reproducibility	Moderate	Moderate	High	High
Robustness	Moderate	Moderate	High	High
Assay time	2–7 d	1–2 d	4–6 h	4–24 h
Sample amount	High	Low	Low	Low

Immunoassays such as ELISA or immunofluorescence offer higher throughput but are costly and only moderately reproducible. Nucleic acid-based assays (e.g., qRT-PCR) provide sensitive and robust viral RNA quantification, yet they often result in higher false-positive rates by detecting non-infectious particles. In contrast, reporter virus-based assays combine high sensitivity, reproducibility, and scalability (up to 1,536-well formats) with moderate cost and shorter assay times (4–24 h). They also generate excellent signal-to-background ratios, particularly when luciferase reporters, which can outperform green-fluorescence protein by up to 1,000 fold (Tannous, 2009; Sfarcic et al., 2019). Nonetheless, false positives may arise from cellular toxicity or interference with reporter expression, underscoring the need for counter-screens. Furthermore, replication-incompetent reporter virus particles (RVPs) have further enhanced safety and reproducibility, particularly for high-containment viruses (Whitbeck et al., 2020).

Recent advances in reverse genetics and recombinant DNA technology have transformed the antiviral discovery landscape by enabling the construction of reporter-expressing viruses. Incorporating fluorescent or bioluminescent reporters into viral genomes allows real-time, quantitative tracking of viral replication with dramatically improved throughput and sensitivity. Reporter viruses therefore bridge the gap between the physiological relevance of phenotype-based assays and the scalability required for modern high-throughput screening (HTS).

A wide range of reporter proteins is now available, each offering distinct advantages. Among them, fluorescent and bioluminescent proteins are especially favored for their superior performance. Their properties have been extensively reviewed (Rowe et al., 2009; Lukyanov, 2022; Vysotski, 2022), as well as their application in virology research both *in vitro* and *in vivo* (Li et al., 2016; Dumm and Heaton, 2019; Lau et al., 2023). This review summarizes the characteristics of currently available fluorescent and bioluminescent proteins, particularly those used in constructing reporter-expressing viruses. It also discusses their applications in antiviral screening, as well as the practical challenges and future directions for this technology.

## 2 Types of reporter molecules for construction of reporter-expressing viruses

Selecting an appropriate reporter protein is critical for developing reporter-expressing viruses for specific applications. A variety of reporter proteins with distinct properties are available, with fluorescent and bioluminescent proteins remaining the most widely used due to their high sensitivity and compatibility with HTS technologies (Nogales et al., 2019).

### 2.1 Fluorescent proteins

Fluorescent proteins (FPs) were first discovered in 1962 when Shimomura and colleagues extracted green fluorescent protein (GFP) from the jellyfish *Aequorea victoria* (Shimomura et al., 1962). Subsequent cloning and expression of GFP in other organisms enabled its use as a molecular reporter (Prasher et al., 1992; Chalfie et al., 1994; Heim et al., 1995). The development of GFP and its variants has become a foundational tool in biological research, earning its inventors the 2008 Nobel Prize in Chemistry (NobelPrize.org, 2008). Thousands of FPs are now cataloged in FPbase, a comprehensive online database (Lambert, 2019).

To effectively use FPs in constructing reporter-expressing viruses, several properties must be considered, including low cytotoxicity against the host cell, exhibiting strong and stable fluorescence, and being resistant to photobleaching, particularly in long-term or time-lapse experiments (Shaner et al., 2005). When used in fusion proteins, they should not oligomerize or interfere with the folding and function of the target protein.

Fluorescence occurs when FPs or dye molecules (e.g., fluorescein, rhodamine) absorb high-intensity light, exciting electrons that emit light at longer wavelengths upon returning to the ground state. However, this process can generate background signals, affecting the signal-to-noise ratio.

Commonly used FPs include red variants (mPlum, Katushka, tdKatuska2, mCherry, mFRP1, mKO), yellow variants (YPet, mCitrine, Venus), and green variants (mNeonGreen, Emerald, eGFP, GFP, ZsGreen, ZsGreen1) (Lambert, 2019). These enable real-time visualization of viral replication and protein expression in HTS assays (Ge et al., 2008; Lee et al., 2013; Zhang et al., 2020), and support multiplexing using distinct emission colors (e.g., eGFP and mCherry) to allow simultaneous monitoring of different viral proteins or co-infections (Rostad et al., 2016).

In addition, FPs have been widely used in *in vivo* studies, where reporter viruses provide insights into viral dynamics and therapeutic efficacy in animal models (Falzarano et al., 2014). **Table 2** summarizes commonly used FPs and their key properties, such as brightness, photostability, oligomerization tendency, and maturation efficiency.

**Table 2:** Fluorescence protein variants commonly used in construction of reporter-expressing viruses. Data is adopted from FPbase (Lambert, 2019).

Protein	Origin	Form	MW (kDa)	Excitation/emission (nm)	Bright-ness^*^	Photosta-bility (s)^**^	Reference
mPlum	*Discosoma* sp. (derived from DsRed)	Monomer	25.6	590/649	4.1	53	(Wang et al., 2004)
Katushka	*Entacmaea quadricolor* (derived from FPS78)	Dimer	26.0	588/635	22	208	(Shcherbo et al., 2007)
tdKatushka2	*Entacmaea quadricolor* (derived from FPS78)	Dimer	26.1	588/633	49	144	(Shcherbo et al., 2009)
mCherry	*Discosoma* sp. (derived from DsRed)	Monomer	26.7	587/610	16	68	(Shaner et al., 2004)
mFRP1	*Discosoma* sp. (derived from DsRed)	Monomer	25.9	584/607	12.5	6.2	(Campbell et al., 2002)
mKO	*Verrillofungia concinna* (derived from KO)	Monomer	24.5	548/559	31	122	(Karasawa et al., 2004)
YPet	*Aequorea victoria* (derived from GFP)	Dimer	26.9	517/530	80	58	(Nguyen and Daugherty, 2005)
mCitrine	*Aequorea victoria* (derived from GFP)	Monomer	27.1	516/529	70	33	(Zacharias et al., 2002)
Venus	*Aequorea victoria* (derived from GFP)	Dimer	26.8	515/528	53	15	(Nagai et al., 2002)
mNeonGreen	*Branchiostoma lanceolatum* (derived from YFP)	Monomer	26.6	506/517	93	158	(Shaner et al., 2013)
Emerald	*Aequorea victoria* (derived from GFP)	Dimer	26.9	487/509	39	0.69	(Cubitt et al., 1998)
GFP	*Aequorea victoria*	Dimer	26.9	395/509	20	NA	(Prasher et al., 1992)
EGFP	*Aequorea victoria* (derived from GFP)	Dimer	26.9	488/507	34	174	(Cormack et al., 1996)
ZsGreen	*Zoanthus* sp.	Tetramer	26.1	493/505	22	NA	(Matz et al., 1999)
ZsGreen1	*Zoanthus* sp. (derived from ZsGreen)	Tetramer	26.1	493/505	NA	NA	

*Molecular brightness is calculated as the product of extinction coefficient and quantum yield.

**Photostability of a fluorescent protein is its ability to resist photobleaching, or the irreversible destruction of a fluorophore by light. Photostability measurements are typically reported as the time it takes for the emission intensity to reach half of its initial value (t_1/2_).

NA, Not available.

Despite their widespread use, classical FPs such as GFP (~27 kDa) may destabilize viral genomes due to their large size. To address this limitation, mini-fluorescence-activating proteins (mini-FAPs) have recently been developed as a new class of compact reporters (~14 kDa) that fluoresce only upon binding to synthetic fluorogens such as DFHBI (3,5-difluoro-4-hydroxybenzylidene imidazolinone) (Klima et al., 2021). Mini-FAPs reduce the genetic footprint, minimize the disruption of viral genome organization and replication, and provide controlled fluorescence activation through exogenous chromophore addition, features that make them ideal for HTS platforms.

Several caveats should be considered when using FPs for *in vivo* imaging. Standard green and yellow FPs exhibit limited tissue penetration and high autofluorescence, reducing sensitivity for deep-tissue applications. To address these limitations, far-red and near-infrared fluorescent proteins (e.g., mKate2, iRFP, mNeptune) have been developed, offering superior penetration and lower background noise, thereby making them more suitable for live-animal imaging (Shcherbo et al., 2007, 2009; Shcherbakova and Verkhusha, 2013). In parallel, advances in tissue-clearing methods such as CLARITY and iDISCO render whole organs optically transparent, enabling three-dimensional visualization of infection and viral dissemination. When incorporated into reporter viruses, these far-red probes expand the utility of such systems beyond cell-based high-throughput antiviral screening (HTS) assays, allowing complementary studies of viral pathogenesis and drug efficacy in intact tissues and animal models. Thus, while compact reporters such as mini-FAPs may be particularly advantageous for HTS applications, the development of far-red reporters and tissue-clearing technologies highlights the broader versatility of reporter virus platforms across both screening and *in vivo* contexts (Eliat et al., 2022; Frenkel et al., 2023).

In summary, the diverse FP toolkit from traditional GFP variants to mini-FAPs and far-red reporters offers powerful molecular probes for constructing reporter-expressing viruses. Integration with advanced imaging approaches extends their utility from *in vitro* HTS assays to *in vivo* pathogenesis and drug efficacy studies (Falzarano et al., 2014).

### 2.2 Bioluminescent proteins

Bioluminescent proteins (BPs) are luciferases that emit light by oxidizing substrates such as luciferin or coelenterazine in the presence of oxygen and, in some cases, ATP (Badr and Tannous, 2011). Found in diverse marine and terrestrial organisms, BPs operate via various mechanisms. For example, *Aequorea victoria* uses calcium-activated photoproteins, while fireflies rely on ATP-dependent luciferases (Haddock et al., 2010). Computational models have revealed the evolutionary diversity and adaptability of BPs (Zhang et al., 2017b, 2020).

Owing to their enzyme-catalyzed light emission and lack of external excitation requirements, BPs provide exceptionally high signal-to-noise ratios by avoiding background noise caused by autofluorescence (Contag and Bachmann, 2002). They also offer deeper tissue penetration and reduced phototoxicity, making them powerful tools for sensitive, quantitative, and non-invasive detection. These advantages have led to their extensive application in molecular imaging detecting titers as low as 10² PFU/mL (Luker et al., 2002), cell-based assays, and the development of reporter-expressing viruses for monitoring viral replication dynamics both *in vitro* and *in vivo*. Their light emission directly reflects enzymatic activity, enabling accurate quantification of viral replication (Luker and Luker, 2010). Additionally, BPs have a broad dynamic range, allowing low and high signal intensities to be detected within the same assay (Branchini et al., 2018).

A notable limitation of BPs is their dependence on external substrates, which must be present in sufficient quantity (Berger et al., 2008). Among the widely used BPs, firefly luciferase (FLuc) is favored for *in vivo* imaging due to the red-shifted emission (~560–620 nm) of its substrate D-luciferin, which improves tissue penetration (Contag et al., 1997; Branchini et al., 2010). Renilla luciferase (RLuc), a bioluminescent enzyme derived from the sea pansy *Renilla reniformis*, emits blue light (~480 nm) upon using coelenterazine. It does not require ATP and is useful for extracellular and multiplexed applications (Lorenz et al., 1991; Loening et al., 2007).

NanoLuc, a luciferase derived from *Oplophorus gracilirostris*, offers ~150-fold higher brightness than FLuc and RLuc, with improved stability (Tran et al., 2013). Although its substrate, furimazine, has suboptimal water solubility and potential cytotoxicity, newer analogs like fluorofurimazine improve solubility and reduce toxicity while maintaining strong luminescence (Smith et al., 2025). These innovations enhance bioluminescence imaging (BLI) reliability for live-cell tracking and animal studies.

Moreover, newly engineered luciferases such as picALuc offer exciting opportunities for next-generation reporter virus systems. This 13 kDa artificial enzyme, substantially smaller than NanoLuc, retains strong activity and remarkable thermostability (Ohmuro-Matsuyama et al., 2022). Rational engineering, including the addition of short charged peptides additions at the C-terminus and targeted point mutations, has enhanced its light output and glow kinetics. Although picALuc has not yet been applied in reporter-expressing viruses, its compact genetic footprint makes it particularly attractive for RNA viruses with strict genome-size constraints, where maintaining replication fitness is challenging. Its small size also facilitates novel applications such as split-luciferase complementation assays, BRET-based detection systems, or dual-reporter constructs with lower fitness costs (Ohmuro-Matsuyama et al., 2023). Future studies validating substrate compatibility, codon optimization, and genomic stability across different viral backbones will determine whether picALuc can become a practical tool in antiviral screening and *in vivo* imaging. **Table 3** lists key BPs and their properties.

**Table 3:** Bioluminescence protein variants commonly used for construction of reporter-expressing viruses.

Protein	Origin	MW (kDa)	Type	Substrate	Co-factor	Signal type	Max light emission (nm)	Relative light Intensity	Reference
Firefly luciferase (FLuc)	Firefly (*Photinus pyralis*)	62	Intra-cellular	Luciferin	ATP	Glow	562-610 (depend on pH)	6.8×10^4^	(de Wet et al., 1985)
Renilla luciferase (RLuc)	Sea pansy (soft corral, *Renilla reniformis*)	35	Intra-cellular	Coelenterazine	None	Flash or glow	475	6.8×10^4^	(Matthews et al., 1977)
Gaussia luciferase (GLuc)	Copepod (*Gaussia princeps*)	18.2	Secreted	Coelenterazine	None	Flash or glow	480	1.7-6.2×10^7^	(Wu et al., 2015)
Nano/NanoKAZ Luciferase (NanoLuc/NanoKAZ)	Deep-sea shrimp (*Oplophorus gracilirostris*)	19.1	Secreted	Furimazine	None	Glow	460	1.7×10^6^	(Hall et al., 2012)
Artificial luciferase (ALuc)	(Inspired by sequence of copepod luciferases)	21	Secreted	Coelenterazine	None	Glow	488	NA	(Kim and Izumi, 2014)
Metridia luciferase (MLuc)	Copepod (*Metridia longa*)	16.5	Secreted	Coelenterazine	None	Glow	488	NA	(Markova et al., 2015)
TurboLuc	Copepod (*Metridia longa*)	16.5	Secreted	Coelenterazine	None	Glow	480	NA	(Auld et al., 2018)
picALuc	(Mutant of ALuc)	13	Secreted	Coelenterazine	None	Glow	488	NA	(Ohmuro-Matsuyama et al., 2022)
HiBiT	(Epitope tag of LgBiT, a 18 kDa NanoLuc derived subunit)	1.3 (11 amino acids)	Intra-cellular/secreted	Fumirazine	None	Glow	460	NA	(Schwinn et al., 2018)

NA, Not available.

## 3 Strategies for reporter-expressing virus construction

Reporter-expressing viruses are typically constructed by inserting a reporter gene into the viral genome at a site that minimally interferes with the RNA’s secondary structure and replication. The insertion site is virus-specific and must be carefully chosen, as improper placement can lead to genome instability, attenuated viral propagation, or loss of the reporter gene during serial passage (Baker and Shi, 2020).

Reporter expression is usually driven by a viral promoter, either through direct fusion with a viral protein or via other regulatory sequences (Li et al., 2016). Because fusion may interfere with protein folding and viral fitness, a 2A cleavage sequence is often included to promote proper protein separation (Luke and Ryan, 2018). Alternatively, inteins-based self-splicing enzymes can enable reporter insertion within open reading frames with minimal structural perturbation (Heiden et al., 2022).

Reporter proteins may be expressed intracellularly or secreted, depending on the presence of secretion motifs. Secreted reporters simplify detection, as activity can be measured directly in the culture supernatant, whereas intracellular reporters require cell lysis, which increases labor and cost (Koutsoudakis et al., 2006; Mulwa et al., 2018; Sandargo et al., 2018; Messaoudi et al., 2022).

In addition to full-length reporters, split-reporter complementation systems such as split-GFP and NanoBiT/HiBiT have been developed to reduce the genetic load on viral genomes while retaining sensitive detection capabilities (Avilov et al., 2012; Dixon et al., 2016; Pannacha et al., 2021). These systems divide the reporter into a small fragment encoded by the virus and a larger complementary fragment provided by the host cell, minimizing genome burden and allowing for robust signal output. However, several limitations constrain their use. First, the requirement for stable cell lines expressing the complementary fragment (e.g., GFP1–10 or LgBiT) reduces flexibility and presents a bottleneck for *in vivo* applications, where generating organisms or animal models stably expressing the larger fragment is often impractical. Second, complementation is not instantaneous and, in the case of split-GFP, the reconstituted fluorophore is essentially irreversible, limiting the monitoring of very rapid, transient, or reversible protein–protein interactions. Third, efficient reconstitution requires high intracellular levels of the complementary fragment, which can be difficult to achieve in certain cell types. Although stable cell lines mitigate this issue, differences in folding efficiency and cellular environment may still lead to weak or inconsistent signals. Finally, as with any protein-tagging strategy, fusion of the reporter fragment to viral proteins can subtly affect their function or replication dynamics, occasionally slowing growth (Avilov et al., 2012). Collectively, while split-reporter systems offer powerful tools for live-cell imaging, their technical and biological constraints must be carefully accounted for when interpreting results.

Recent innovations have expanded reporter virus design. Alphaviruses, for instance, tolerate insertions in the hypervariable domain of nsP3 without impairing replication (Kümmerer et al., 2012; Santos et al., 2021), while picornaviruses accommodate reporters at the P1-P2 junction or VP4 N-terminus (Bakhache et al., 2025). These highlight that reporter insertion “hotspots” can be family-specific, reflecting differences in genome organization. Deep insertional mutagenesis and scanning now allow systematic mapping of genome regions that tolerate exogenous sequences (Bakhache et al., 2025), providing a more rational framework for engineering reporter viruses and potentially reducing reliance on trial-and-error design. Ultimately, the choice of reporter also affects stability. Compact reporters such as NanoLuc (19 kDa), picALuc (13 kDa), or mini-FAPs (14 kDa) impose less genome burden then conventional GFP (27 kDa), improving the propagation stability in RNA viruses with strict size constraints (de Wet et al., 1985; Markova et al., 2015; Klima et al., 2021; Ohmuro-Matsuyama et al., 2022).

The following sections summarize insertion sites and expression strategies across different viral families. Some viral families are grouped together due to the limited number of reporter-expressing virus examples available for them.

### 3.1 Flaviviridae

*Flaviviridae* is a family of enveloped, positive-sense, single-stranded RNA viruses (9–13 kb). The 3’ end forms a loop structure, while the 5’ end has either a methylated cap or a genome-linked protein (VPg) to initiate translation (Simmonds et al., 2017).

**Figure 1:**
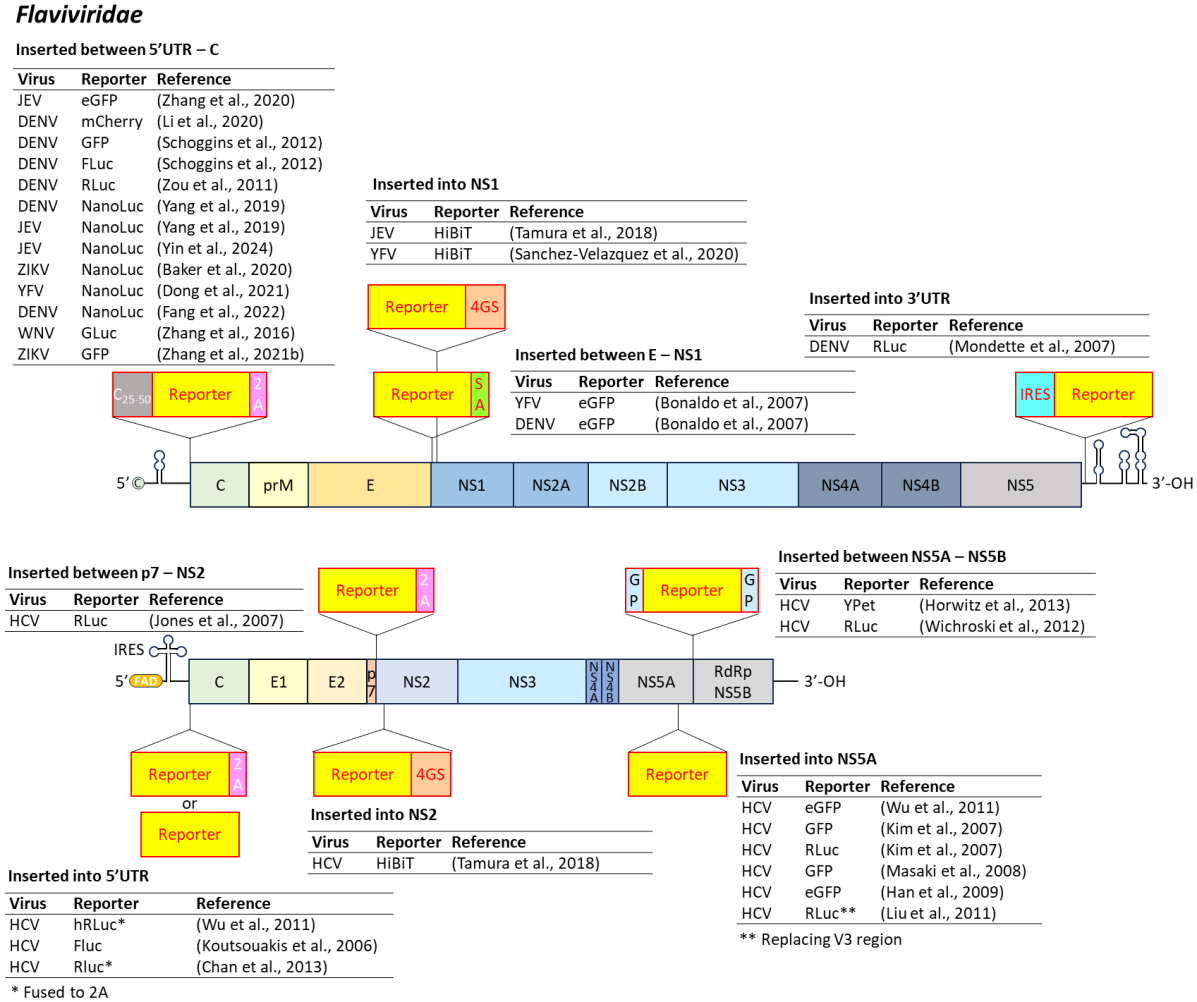
Insertion position of reporter-coding genes in the genome of several viruses in the family *Flaviviridae*. JEV, Japanese encephalitis virus; DENV, dengue virus; ZIKV, zika virus; YFV, yellow fever virus; WNV, West Nile virus; HCV, hepatitis C virus; IRES, internal ribosome entry site; 2A, 2A peptides; SA, stem-anchor sequences; 4GS, 4GS linker sequences; GP, glycine-proline linker sequences; FAD, flavin adenine dinucleotide.

Reporter gene insertion in the 5’UTR has been widely applied in *Flavivirus* constructs (**Figure 1**). Capsid gene duplication is often included to stabilize recombinant viruses, with lengths ranging from 25 to 50 amino acids; 38 residues have been reported optimal for ZIKV (Baker et al., 2020). Incorporating the DCS-PK pseudoknot element further improves replication (Liu et al., 2013). A 2A peptide is commonly added to the C-terminus of the reporter gene to facilitate cleavage and prevent interference with the core protein. This strategy has enabled stable incorporation of various reporters: NanoLuc in DENV, JEV, ZIKV, and YFV (Yang et al., 2019; Baker et al., 2020; Dong et al., 2021; Fang et al., 2022; Yin et al., 2024), FLuc in DENV (Schoggins et al., 2012), GLuc in WNV (Zhang et al., 2016), RLuc in DENV (Zou et al., 2011), GFP in DENV and ZIKV (Schoggins et al., 2012; Zhang et al., 2021), eGFP in JEV (Zhang et al., 2020), and mCherry in DENV (Li et al., 2020a).

Other strategies include inserting eGFP between E and NS1 genes in YFV and DENV, using ER signal peptides and stem-anchor sequences to direct proper protein localization and cleavage (Bonaldo et al., 2007). The small HiBiT tag has also been inserted into NS1 (JEV/YFV) and NS2 (HCV) to minimize structural disruption while enabling detection upon LgBiT addition (Tamura et al., 2018; Sanchez-Velazquez et al., 2020). Insertion into the 3’UTR of DENV has also been used, incorporating an encephalomyocarditis virus internal ribosome entry site (EMCV IRES) to regulate reporter gene translation (Mondotte et al., 2007).

HCV genomes have been engineered similarly. Reporter genes have been inserted into the 5’UTR, between p7 and NS2, NS5A, and between NS5A and NS5B (Koutsoudakis et al., 2006; Jones et al., 2007; Kim et al., 2007; Masaki et al., 2008; Han et al., 2009; Liu et al., 2011; Wu et al., 2011; Wichroski et al., 2012; Chan et al., 2013; Horwitz et al., 2013). Stability has been enhanced using 2A peptides or fusion with the gateway peptide spanning NS3–NS4A cleavage sites (Pietschmann et al., 2006).

### 3.2 Filoviridae

*Filoviridae* is a family of negative-sense, single-stranded RNA viruses (15–19 kb), including Ebola virus (EBOV) and Marburg virus (MARV). These pathogens are responsible for severe hemorrhagic fevers with high mortality rates, particularly in West Africa (Feldmann and Geisbert, 2011; Abir et al., 2022). Due to the high risk these viruses pose, work with these viruses generally requires BSL-4 containment, making antiviral research challenging.

**Figure 2:**
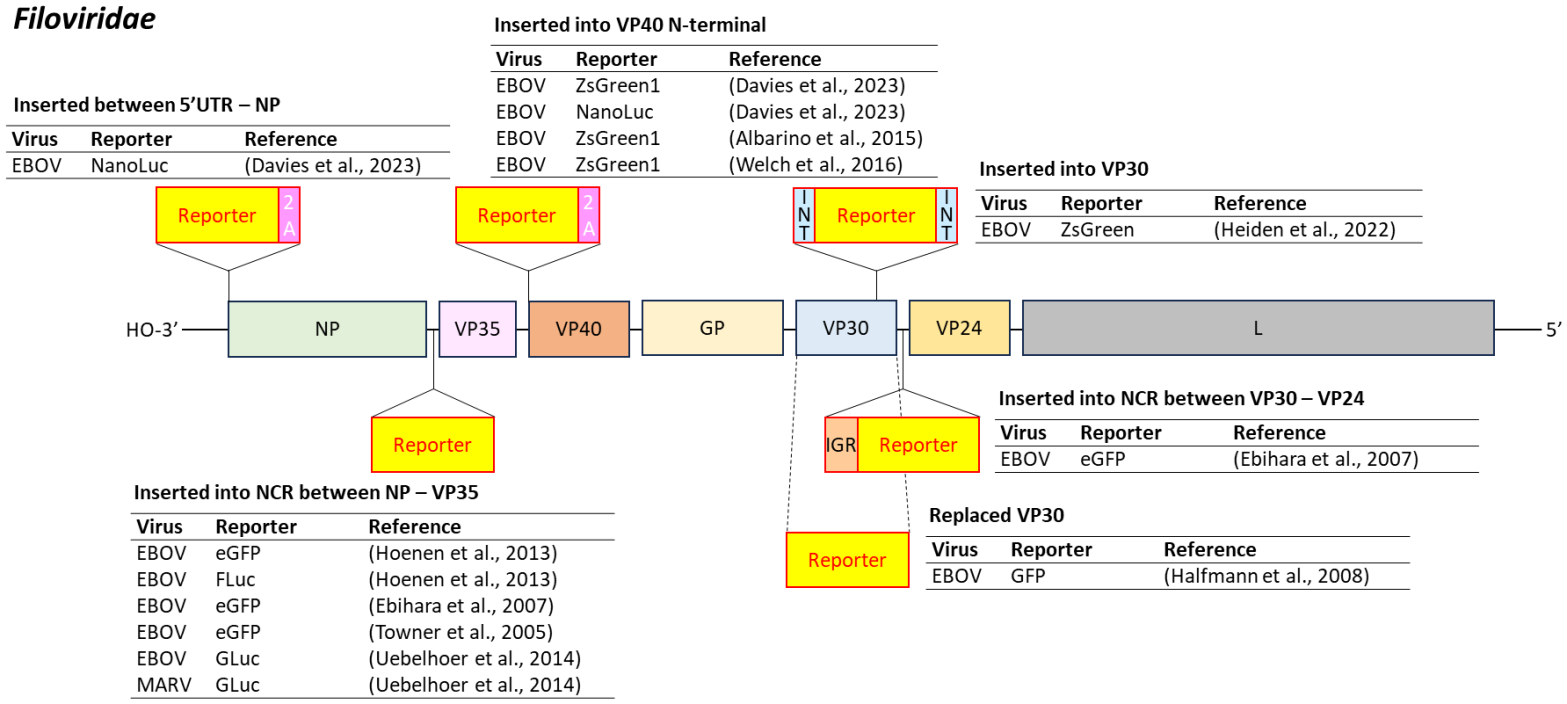
Insertion position of reporter-coding genes in the genome of several viruses in the family *Filoviridae*. EBOV, Ebola virus; MARV, Marburg virus; 2A, 2A peptides; INT, inteins; IGR, intergenic region. Dashed line represents replacement of the target gene by the reporter gene.

Reporter-expressing versions of EBOV and MARV have been extensively developed to improve the safety and efficiency of antiviral screening. Common insertion sites for reporter genes include the region between NP and VP35, which has supported expression of eGFP, GLuc, and FLuc in replication-competent constructs (Towner et al., 2005; Ebihara et al., 2007; Hoenen et al., 2013; Uebelhoer et al., 2014) (**Figure 2**).

Another strategy targets the 5’ end of the VP40 gene, incorporating reporter genes such as ZsGreen1 and NanoLuc via a 2A peptide (Albariño et al., 2015; Welch et al., 2016; Davies et al., 2023). Additionally, the 5’UTR of EBOV has proven suitable for constructing NanoLuc-expressing, replication-competent viruses (Davies et al., 2023).

Reporter genes have also been inserted between VP30 and VP24, with an intergenic region (IGR) placed upstream of the reporter to mimic natural gene expression (Ebihara et al., 2007). In another approach, the VP30 gene, which encodes a transcription factor, was replaced with a reporter gene. This virus replicates efficiently only in Vero cells engineered to express VP30, producing titers comparable to wild-type EBOV (Halfmann et al., 2008). Although biologically contained, the virus must still be handled under appropriate biosafety levels due to its residual infectious potential.

Inteins have also been used to splice reporter proteins into VP30 without disrupting viral replication. This approach demonstrated efficient expression of the reporter gene while preserving viral fitness (Heiden et al., 2022).

### 3.3 Hepeviridae

*Hepeviridae* is a family of non- or quasi-enveloped, positive-sense, single-stranded RNA viruses (6.4–7.2 kb). The genome features a 5’ cap and a polyadenylated 3’ end, allowing it to function both as genomic RNA and mRNA (Purdy et al., 2022).

Reporter gene insertion sites in the hepatitis E virus (HEV) genome include the hypervariable region (HVR) and ORF2. Natural insertions into the HVR have been observed in HEV strains isolated from infected humans and swine (Peralta et al., 2009; Takeuchi et al., 2015). Reporter-expressing HEV constructs incorporating NanoLuc or NanoKAZ at this site have maintained replication competence (Szkolnicka et al., 2019; Primadharsini et al., 2022).

**Figure 3:**
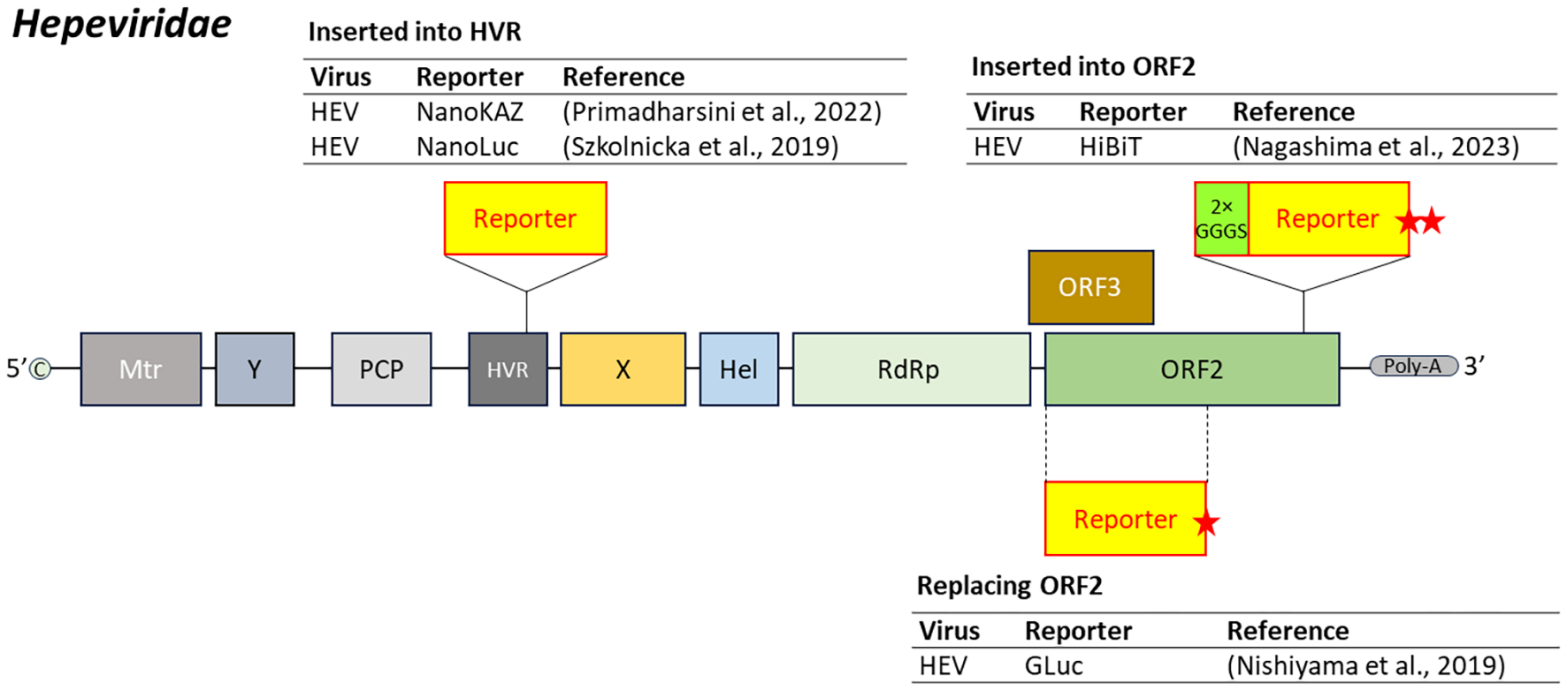
Insertion position of reporter-coding genes in the genome of virus in the family *Hepeviridae*. HEV, hepatitis E virus; 2×GGGS, 2×GGGS linker sequences. Red star mark represents stop codon. Dash line represents replacement of the region by the reporter gene.

Another strategy targets ORF2, which encodes the viral capsid protein. In one approach, the start codon of ORF3 is disrupted and a reporter gene is inserted into ORF2, replacing a 585-base-pair fragment at the 5’ end with a stop codon (Nishiyama et al., 2019). Alternatively, the reporter gene can be inserted at the 3’ end of ORF2, flanked by a tandem glycine-serine linker (2×GGGS) at the N-terminus and two stop codons at the C-terminus (Nagashima et al., 2023) (**Figure 3**).

### 3.4 Togaviridae

*Togaviridae* are enveloped, positive-sense, single-stranded RNA viruses (10–12 kb) with genomes capped at the 5’ end and polyadenylated at the 3’ end (Chen et al., 2018). Chikungunya virus (CHIKV), a member of this family, is a human pathogen that shares structural similarities with Semliki Forest virus (SFV).

**Figure 4:**
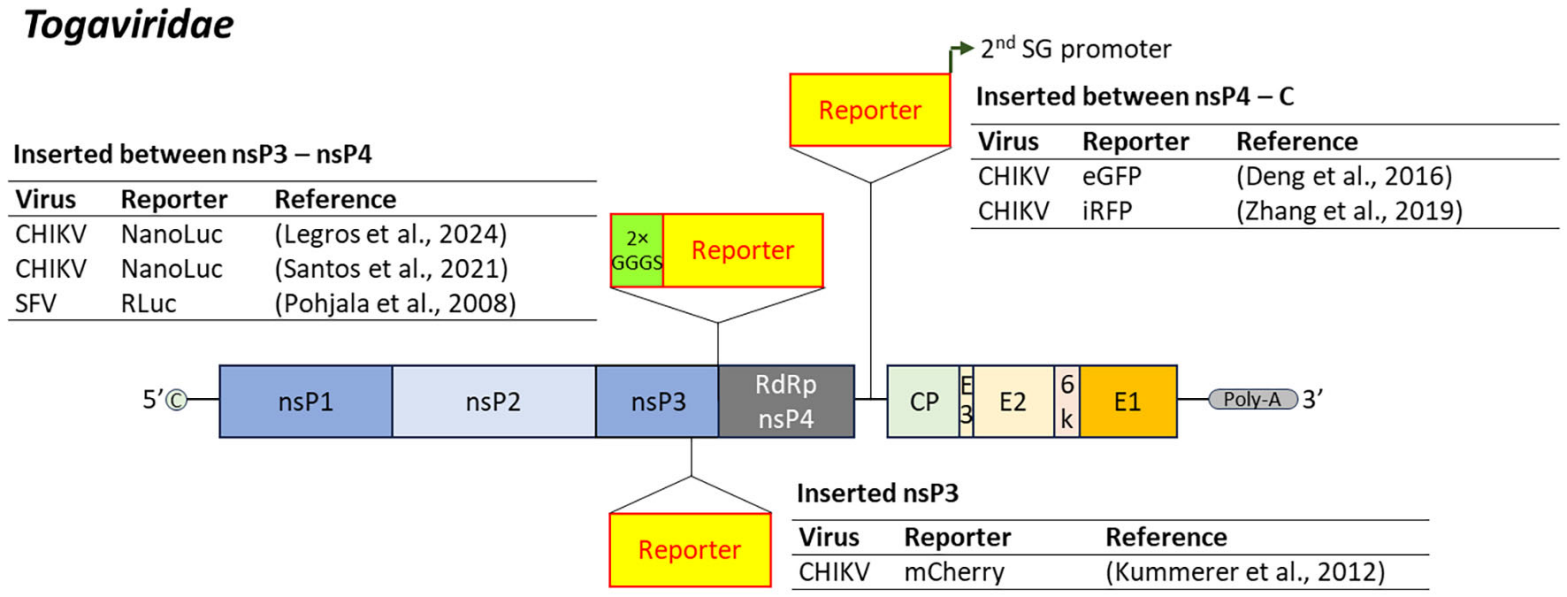
Insertion position of reporter-coding genes in the genome of virus in the family *Togaviridae*. CHIKV, chikungunya virus; SFV, Semliki Forest virus; 2×GGGS, 2×GGGS linker sequences; SG promoter, subgenomic promoter.

Reporter-expressing CHIKV and SFV have been developed by inserting reporter genes at specific sites within the viral genome. A common strategy is to insert the reporter between nsP3 and nsP4, using a double glycine-serine (Gly-Ser) linker to facilitate expression (Pohjala et al., 2008; Santos et al., 2021; Legros et al., 2024). Alternatively, insertion between nsP4 and the capsid gene with a secondary subgenomic promoter downstream maintains expression of the structural proteins (Deng et al., 2016; Zhang et al., 2019). Insertion at the 3’ end of nsP3 has also yielded stable recombinant CHIKV constructs, retaining growth characteristics comparable to the parental strain (Kümmerer et al., 2012) (**Figure 4**).

### 3.5 Coronaviridae

*Coronaviridae* are enveloped, positive-sense, single-stranded RNA viruses (22–36 kb). Their genome is capped at the 5’ end and polyadenylated at the 3’ end. Members of the genus *Betacoronavirus*, including SARS-CoV (2002), MERS-CoV (2012), and SARS-CoV-2 (2019), have caused major global outbreaks, highlighting their global health relevance (Woo et al., 2023).

**Figure 5:**
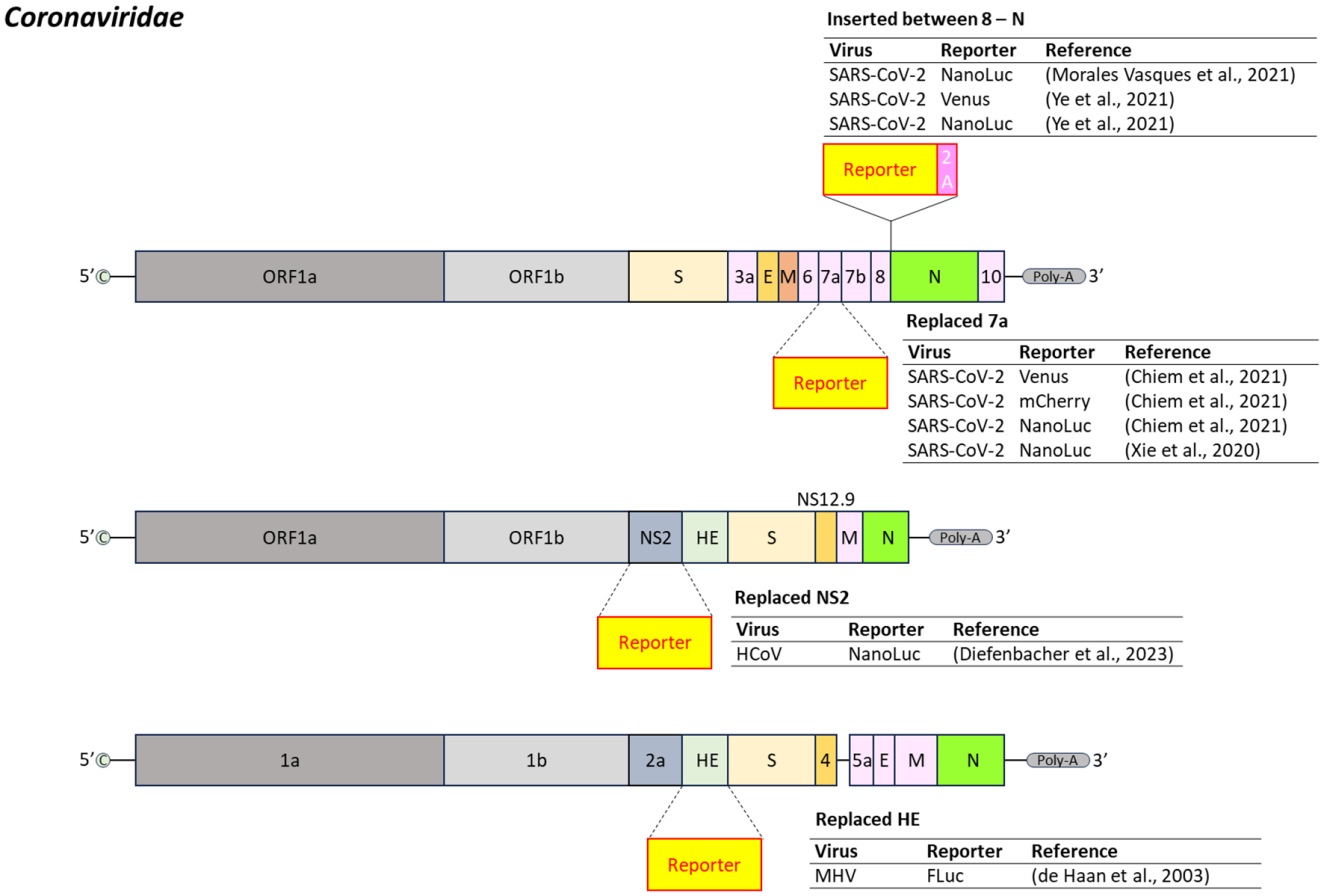
Insertion position of reporter-coding genes in the genome of virus in the family *Coronaviridae*. SARS-CoV-2, severe acute respiratory syndrome corona virus 2; HCoV, human coronavirus; MHV, mouse hepatitis virus (murine coronavirus); 2A, 2A peptides. Dash line represents replacement of the region by reporter gene.

The COVID-19 pandemic spurred extensive development of reporter-expressing SARS-CoV-2 constructs (**Figure 5**). One common strategy involves inserting a reporter gene between ORF8 and the Nucleocapsid (N) gene region, which overlaps with ORF9b (Morales Vasquez et al., 2021; Ye et al., 2021). Another approach replaces the accessory gene ORF7a with the reporter gene, enabling the successful generation of various reporter-expressing SARS-CoV-2 variants (Xie et al., 2020; Chiem et al., 2021).

For human coronavirus OC43 (HCoV-OC43), a seasonal respiratory virus, a NanoLuc-expressing version was engineered by replacing the NS2 accessory gene. Reporter gene expression was controlled by a T7 promoter (Diefenbacher et al., 2024).

Reporter gene insertion site strongly influences stability. In murine hepatitis virus (MHV), RLuc was stably expressed from multiple genome positions, whereas FLuc was prone to deletions within its expression cassette (de Haan et al., 2003) (**Figure 5**).

### 3.6 Pneumoviridae

*Pneumoviridae* are enveloped, negative-sense, single-stranded RNA viruses (13.2–15.3 kb) (Rima et al., 2017). Respiratory syncytial virus (RSV), a major human pathogen, primarily affects infants and causes infections in the nose, throat, and lungs.

**Figure 6:**
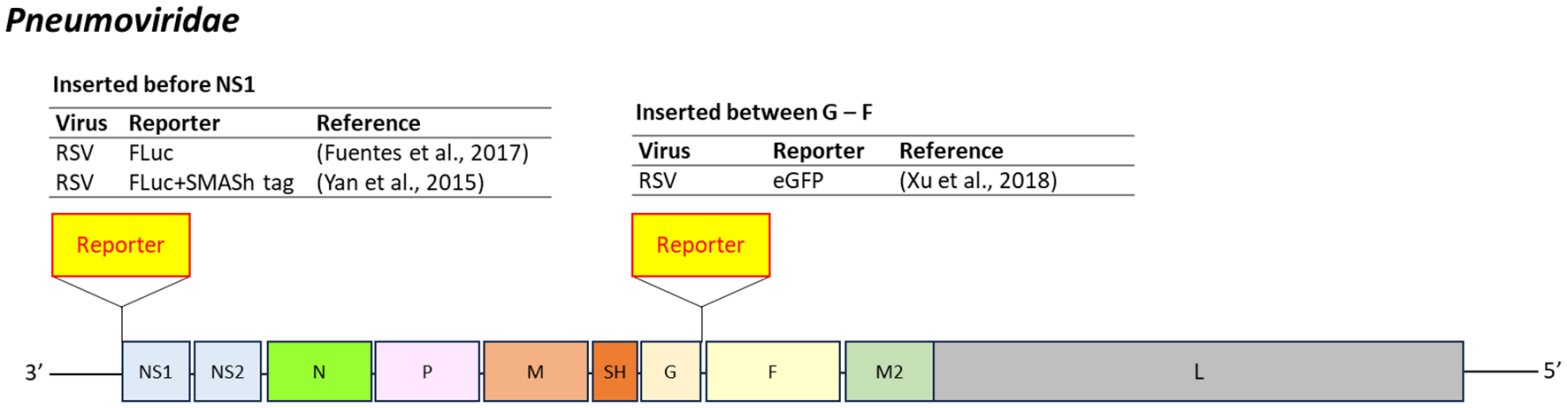
Insertion position of reporter-coding genes in the genome of virus in the family Pneumoviridae. RSV, respiratory syncytial virus; SMASh, small molecule-assisted shutoff (Chung et al., 2015).

Reporter-expressing RSV has been developed with reporter insertions at two main sites: upstream of NS1 and between the G and F genes (**Figure 6**). FLuc has been inserted before NS1 to facilitate live imaging studies (Fuentes et al., 2017). Another construct combined FLuc with the small molecule-assisted shutoff (SMASh) system, which employs the HCV-derived NS3 protease domain linked to a degron for post-translational degradation control. During viral stock preparation, the addition of a protease inhibitor (e.g., ASV) prevents cleavage and ensures the degradation of residual reporter proteins (Chung et al., 2015; Yan et al., 2015). Although FLuc activity was reduced by 90%, SMASh enhanced assay performance with a 23-fold increase in signal-to-noise ratio.

In another strategy, a reporter gene was inserted between the G and F genes of RSV, driven by a T7 promoter-expressing cell line. The recombinant virus remained stable for at least nine passages but exhibited slightly reduced growth relative to the wild-type strain (Xu et al., 2018) (**Figure 6**).

### 3.7 Orthomyxoviridae

*Orthomyxoviridae* is a family of negative-sense, single-stranded RNA viruses with segmented genomes (6–8 segments, 10.0–14.6 kb) (Kuhn et al., 2023). Influenza A virus (IAV), the most extensively studied member, is classified based on two surface glycoproteins: hemagglutinin (HA or H) and neuraminidase (NA or N). Notable IAV strains include H1N1 (Spanish flu in 1918, swine flu in 2009) and H5N1 (bird flu).

Reporter-expressing IAV constructs have primarily targeted segment 8, which encodes non-structural proteins, for reporter gene insertion. Typically, GFP or luciferase is fused to the C-terminus of NS1 with or without a 2A peptide to promote separation (Manicassamy et al., 2010; Perez et al., 2013; Eckert et al., 2014; Nogales et al., 2015; Reuther et al., 2015; Probst et al., 2023; Zhang et al., 2023a).

**Figure 7:**
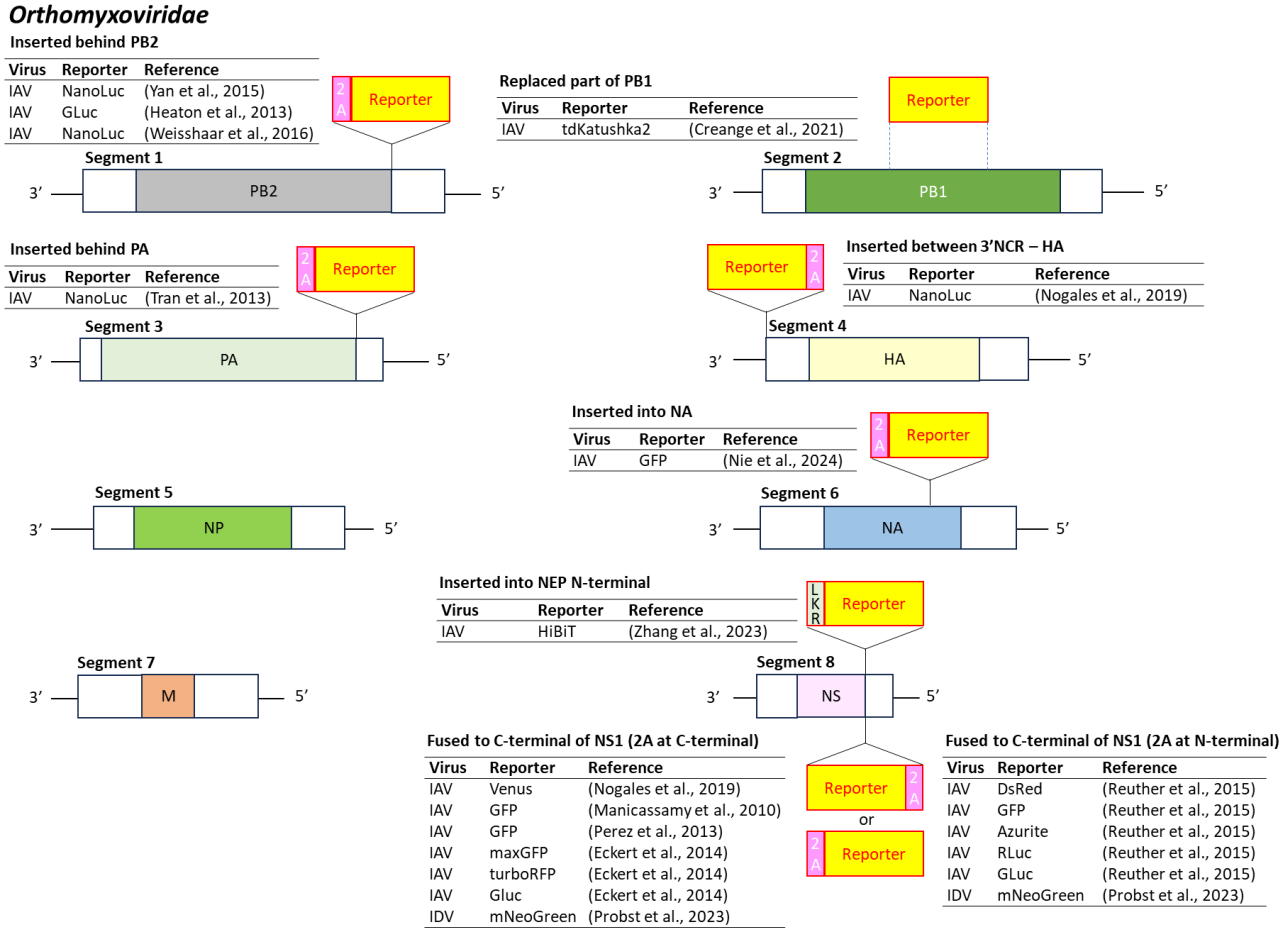
Insertion position of reporter-coding genes in the genome of virus in the family *Orthomyxoviridae*. IAV, influenza A virus; IDV, influenza D virus; 2A, 2A peptides; LKR, linker. White boxes indicate the packaging signals that are responsible for efficient encapsidation into nascent virion. Dashed line represents replacement of the target gene by the reporter gene.

Reporter insertions have also been explored in other segments. The 5’ end of PB2 (segment 1) has supported insertion of reporter-2A fusions (Heaton et al., 2013; Yan et al., 2015; Weisshaar et al., 2016), and segment 3 (PA) has been modified similarly (Tran et al., 2013). Insertion into the 3’ end of segment 4 (HA) (Nogales et al., 2015) and segment 6 (NA) (Nie et al., 2024) has also been successful. Furthermore, partial replacement of the PB1 coding region (segment 2) with a reporter gene has yielded functional reporter-expressing IAVs (Creanga et al., 2021) (**Figure 7**).

### 3.8 Retroviridae

*Retroviridae* is a family of viruses that contain two copies of positive-sense, single-stranded RNA (7–13 kb), each capped at the 5’ end and polyadenylated at the 3’ end (Coffin et al., 2021). The most well-known member is the human immunodeficiency virus (HIV), which causes acquired immunodeficiency syndrome (AIDS).

**Figure 8:**
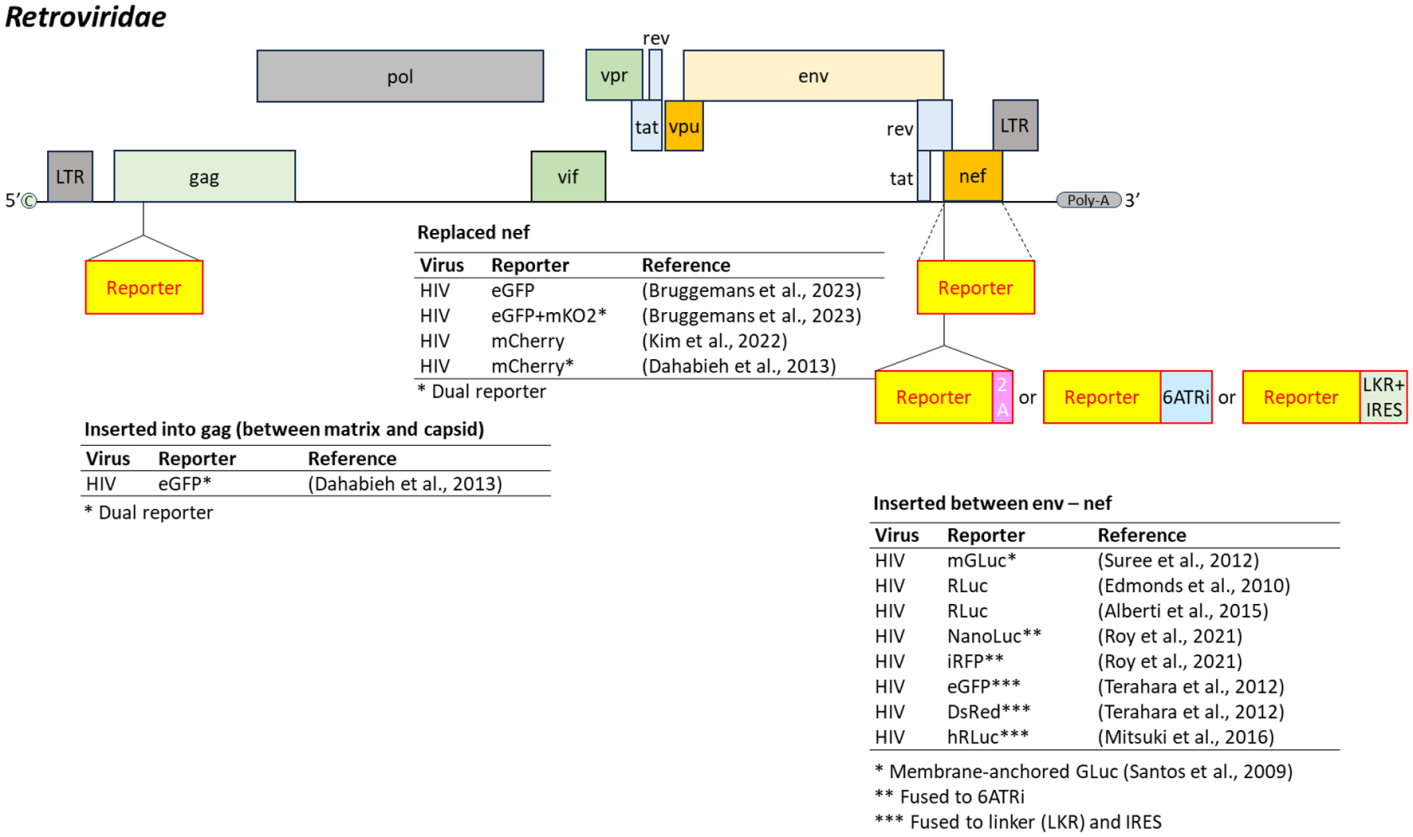
Insertion position of reporter-coding genes in the genome of virus in the family *Retroviridae*. HIV, human immunodeficiency virus; 2A, 2A peptides; 6ATRi, modified 6×AT-rich inhibitory sequence; LKR, linker; IRES, internal ribosome entry site. Dashed line represents replacement of the target gene by the reporter gene.

Reporter-expressing HIV-1 constructs commonly target the *nef* gene, a multifunctional accessory protein that post-transcriptionally mediates CD4 and MHC-I downregulation, modulates host immune signaling, enhances infectivity, and counteracts restriction factors such as SERINC3 and SERINC5 (Roeth and Collins, 2006) (**Figure 8**). One approach inserts a reporter gene at the 5’ end of *nef*, flanked by a 2A peptide and driven by the LTR (Edmonds et al., 2010; Alberti et al., 2015). In another strategy, a CD8 transmembrane fusion anchors the reporter to the membrane for detecting infected cells and viral particles (Suree et al., 2012).

Some constructs incorporate internal ribosome entry site (IRES), such as 6ATRi (a modified 6×AT-rich inhibitory sequence) or the encephalomyocarditis virus IRES, to allow co-expression of Nef and the reporter (Terahara et al., 2012; Mitsuki et al., 2016; Roy et al., 2021). Alternatively, the *nef* gene can be completely replaced with a reporter gene to enable monitoring of latent infection (Kim et al., 2022; Bruggemans et al., 2023). In cell culture, truncation or replacement of *nef* does not impact immune modulation, but impairs replication efficiency and progeny infectivity, a major limitation for HIV-1 reporter assays.

Dual-reporter HIV-1 systems combining a *gag*-encoded reporter with *nef* replacement were developed to differentiate latent versus active infection states (Dahabieh et al., 2013). Importantly, Dahabieh and colleagues adapted this system for high-throughput screening of latency-reversing agents, underscoring its utility in specialized drug discovery. Nevertheless, for routine antiviral screening targeting viral replication, simple single-reporter constructs remain preferred due to their reduced genetic complexity and higher stability (**Figure 8**).

### 3.9 Picornaviridae

*Picornaviridae* includes non-enveloped positive-sense single-stranded RNA viruses (6.7–10.1 kb) that infect diverse vertebrates and invertebrates (Zell et al., 2017). Enterovirus 71 (EV71) and coxsackievirus (COXSV) are notable human pathogens, with COXSV causing myocarditis and meningoencephalitis, particularly in neonates (Chang, 2008; Sin et al., 2015).

**Figure 9:**
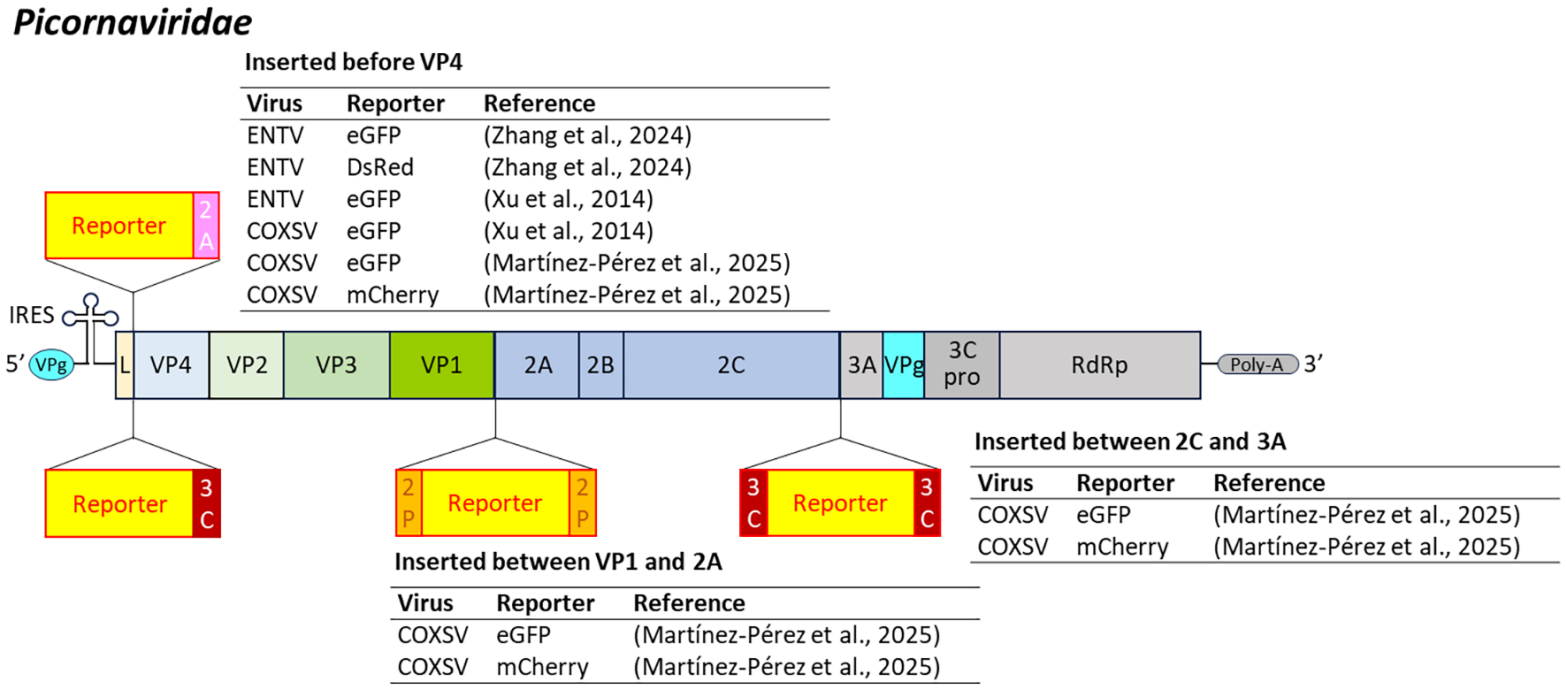
Insertion position of reporter-coding genes in the genome of virus in the family *Picornaviridae*. ENTV, enterovirus; COXSV, coxsackievirus; 2A, 2A peptides; 3C, 3C protease cut site; 2P, 2A protease cut site.

For enteroviruses, reporter-expressing ENTV and COXSV were developed by inserting the reporter gene in the upstream of the VP4 regions, flanked by an EV71 2A protease cleavage site to ensure proper protein separation (Xu et al., 2014; Zhang et al., 2024) or the COXSV 3C protease cut site (Martínez-Pérez et al., 2025). Additional reporter-expressing COXSV constructs carried the reporter gene at the junction of P1-P2 (between VP1 and 2A) and P2-P3 (between 2C and 3A). These recombinant COXSVs were successfully rescued in HEK293T cells, and fluorescence was detectable in all but the P1-P2 eGFP construct (**Figure 9**), underscoring the impact of the reporter sequence on viral fitness (Martínez-Pérez et al., 2025).

### 3.10 *Arenaviridae* and *Nairoviridae*

*Arenaviridae* is a family of segmented, negative-sense single-stranded RNA viruses containing two or three genome segments. Hosts include fish (antennaviruses), mammals (mammarenaviruses), and reptiles (hartmaniviruses and reptarenaviruses) (Radoshitzky et al., 2023). Lassa virus (LASV), a mammarenavirus, causes Lassa fever, a hemorrhagic disease endemic to West Africa, affecting an estimated 500,000 people and resulting in 10,000 deaths annually (Asogun et al., 2019). Lujo virus (LUJV), another highly pathogenic member, was identified during a 2008 outbreak in Southern Africa involving only five documented cases (Simulundu et al., 2016).

*Nairoviridae* comprises enveloped, segmented negative-sense single-stranded RNA viruses with two or three genome segments (17.1–22.8 kb). Hosts include birds, humans, rodents, bats, ticks, and other animals (Garrison et al., 2020). The most notable member, Crimean-Congo hemorrhagic fever virus (CCHFV), causes severe febrile illness and was first reported in the 1960s in Crimea and Congo (Casals, 1969). The virus is primarily transmitted by *Hyalomma* ticks (Papa et al., 2017).

**Figure 10:**
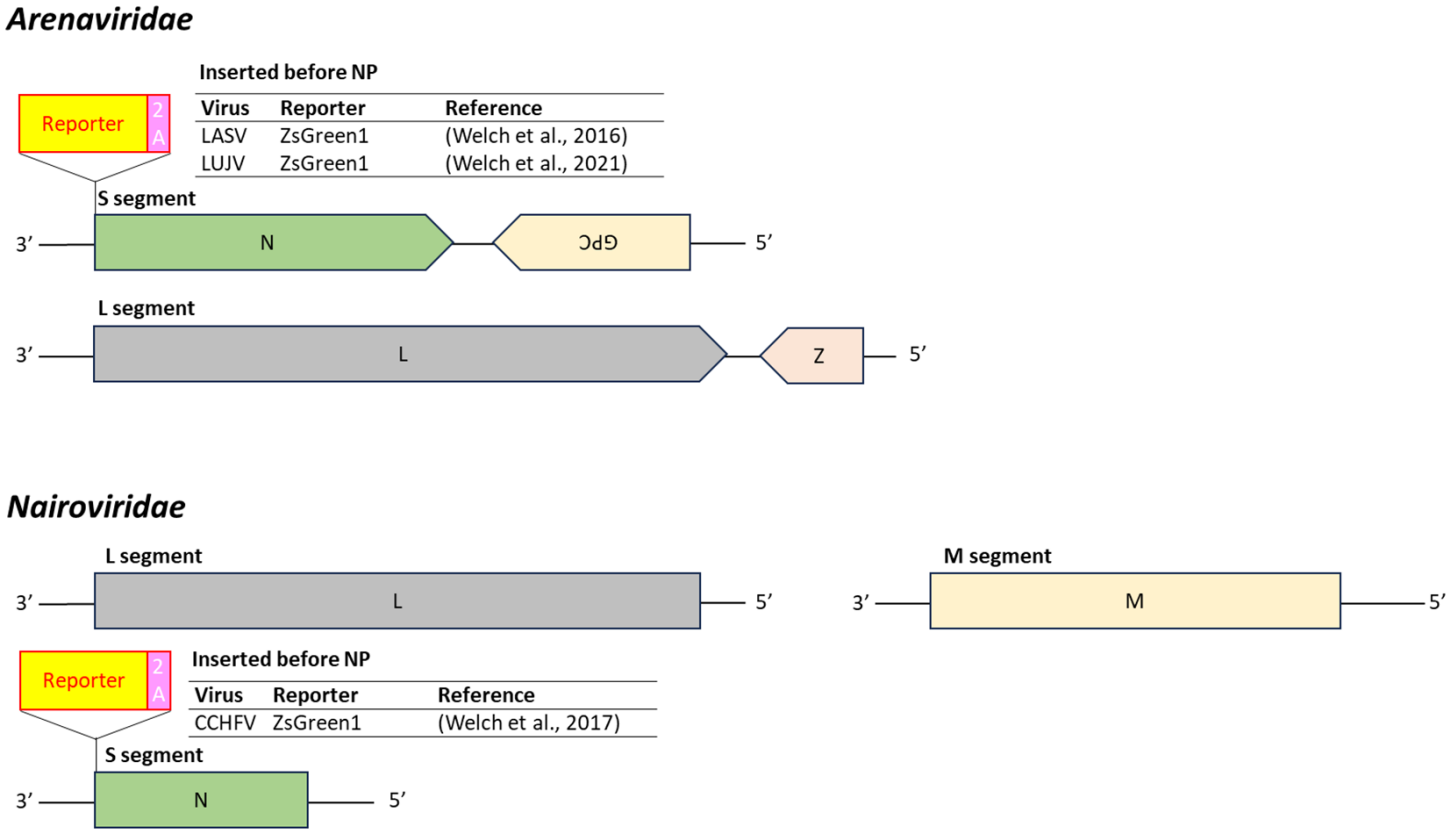
Insertion position of reporter-coding genes in the genome of virus in the family *Arenaviridae* and *Nairoviridae*. LASV, lassa virus; LUJV, lujo virus; CCHFV, Crimean-Congo hemorrhagic fever virus; 2A, 2A peptides.

Reporter-expressing versions of LASV, LUJV, and CCHFV have been engineered by inserting reporter genes upstream of the *N* gene in the S segment. These constructs typically use a 2A peptide to separate the reporter from the viral protein, minimizing structural interference and maintaining viral function (Welch et al., 2016, 2017, 2021) (**Figure 10**).

### 3.11 *Phenuiviridae* and *Peribunyaviridae*

*Phenuiviridae* are enveloped, segmented negative-sense single-stranded RNA viruses containing two to eight genome segments (8.1–25.1 kb). Members of this family infect a wide range of hosts, including vertebrates, invertebrates, plants, and fungi (Sasaya et al., 2023). Rift Valley fever virus (RVFV), a medically significant member, was first discovered in Kenya in the 1930s and is now endemic in sub-Saharan Africa and the Arabian Peninsula. RVFV primarily infects livestock and is transmitted to humans via mosquito bites, contact with infected animal fluids, inhalation of aerosols in slaughterhouses, or consumption of raw milk (Wright et al., 2019).

*Peribunyaviridae* include enveloped, segmented negative-sense single-stranded RNA viruses containing two to eight genome segments (10.7–12.5 kb). Hosts include a variety of vertebrates and invertebrates, such as mammals, birds, mosquitoes, and sandflies (de Souza et al., 2024). Ebinur Lake virus (EBIV), identified in 2014 in Xinjiang, China, can infect cell lines from multiple species, indicating its potential as a zoonotic pathogen (Xia et al., 2020).

**Figure 11:**
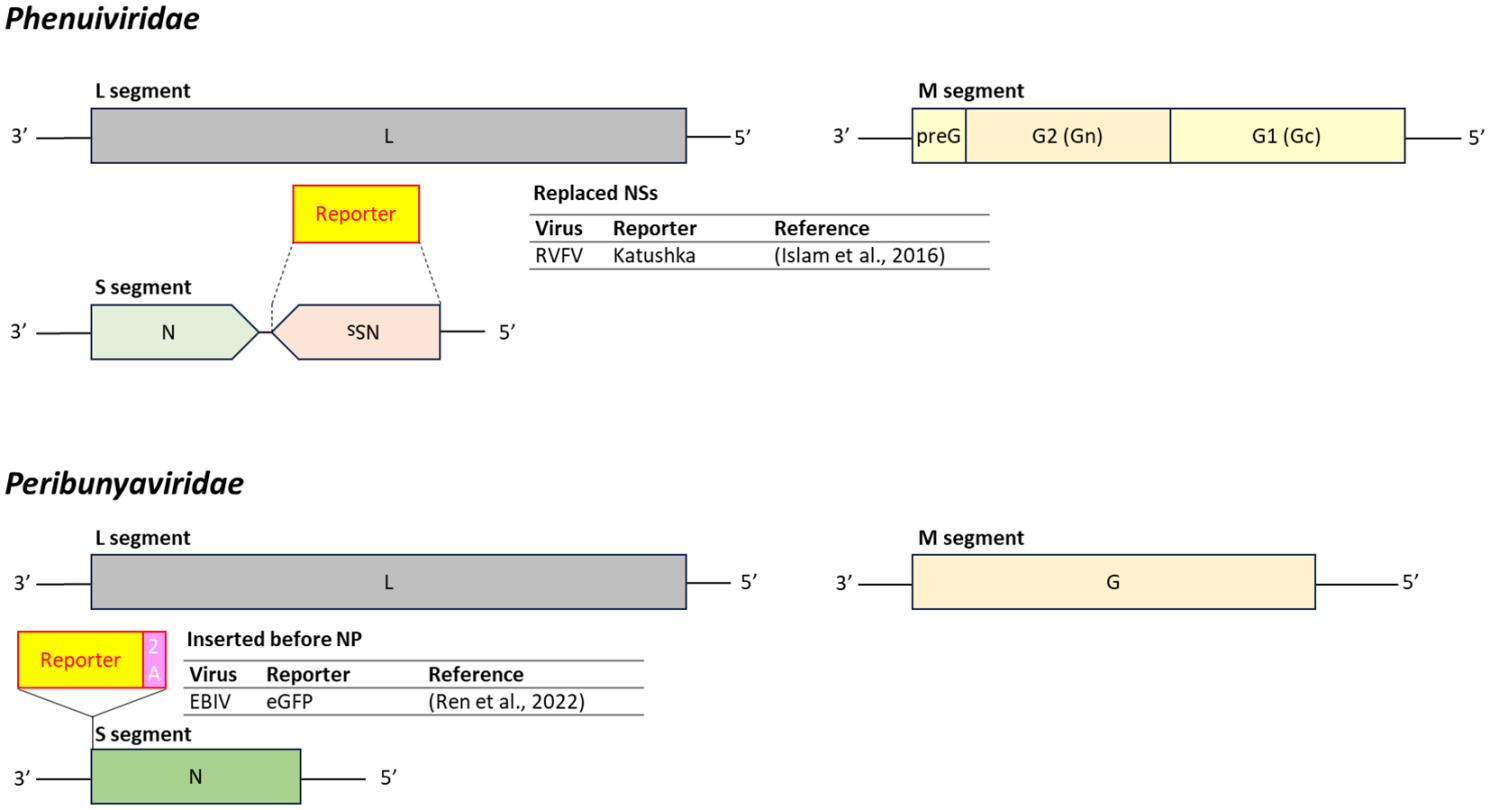
Insertion position of reporter-coding genes in the genome of virus in the family *Phenuiviridae* and *Peribunyaviridae*. RVFV, Rift Valley fever virus; EBIV, Ebinur Lake orthobunyavirus; 2A, 2A peptides. Dashed line represents replacement of the target gene by the reporter gene.

Reporter-expressing RVFV has been developed by replacing the virulence-associated *NSs* gene in the S segment with a reporter gene, allowing the virus to be handled under BSL-2 conditions (Islam et al., 2016). For EBIV, the reporter gene was inserted upstream of the *N* gene in the S segment and separated using the porcine teschovirus-1 2A peptide, ensuring expression of both proteins without structural interference (Ren et al., 2022) (**Figure 11**).

### 3.12 *Paramyxoviridae* and *Adenoviridae*

*Paramyxoviridae* comprises enveloped negative-sense single-stranded RNA viruses (~15 kb) that infect mammals, birds, fish, and reptiles (Rima et al., 2019). Parainfluenza virus (PIV) causes lower respiratory tract infections in children, while Sosuga virus (SOSV), discovered in 2012 in a biologist returning from South Sudan and Uganda, is a zoonotic pathogen (Markarian and Abrahamyan, 2024).

*Adenoviridae* are non-enveloped double-stranded DNA viruses (25–48 kb) that infect mammals, birds, reptiles, amphibians, and fish (Benkő et al., 2022). Human adenoviruses (HADVs) are clinically significant, especially in children and immunocompromised patients (Louie et al., 2008).

**Figure 12:**
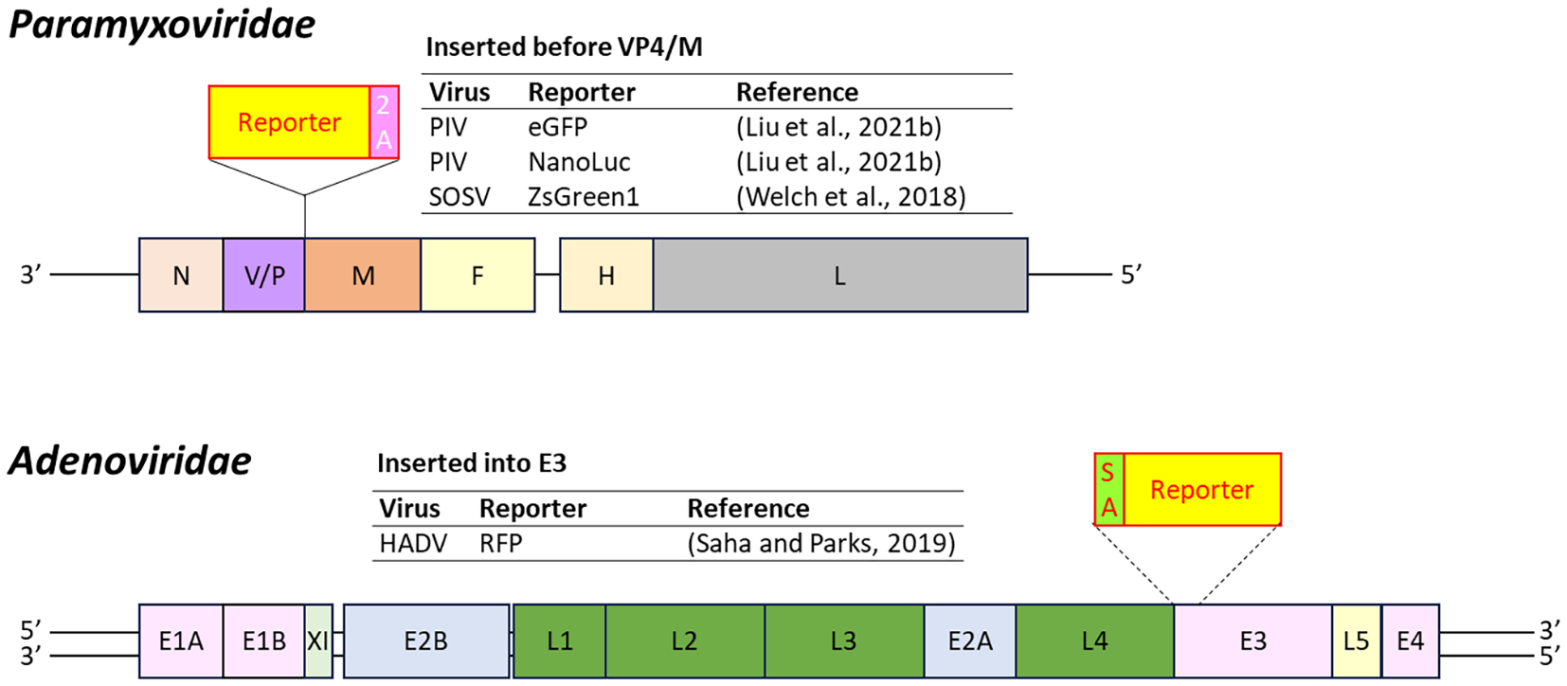
Insertion position of reporter-coding genes in the genome of virus in the family *Paramyxoviridae* and *Adenoviridae*. PIV, parainfluenza virus; SOSV, sosuga virus; HADV, human adenovirus; 2A, 2A peptides; SA, splice acceptor site. Dashed line represents replacement of the target gene by the reporter gene.

Reporter-expressing PIV and SOSV were developed by inserting the reporter gene between the *VP4* and *M* genes (Welch et al., 2018; Liu et al., 2021a, b). RFP-expressing HADV was engineered by replacing the E4 region with an *RFP* gene preceded by a splice acceptor site (Saha and Parks, 2019) (**Figure 12**).

In conclusion, while many strategies have been established to construct reporter-expressing viruses, the optimal design must balance genetic stability, viral fitness, and signal strength. Family-specific hotspots such as the nsP3 hypervariable domain in alphaviruses and the L–VP4 junction in picornaviruses illustrate that insertion strategies are highly context-dependent. The integration of modern tools like deep insertional mutagenesis with the use of smaller reporters (e.g., NanoLuc, picALuc, mini-FAPs) provides a more rational path toward genetically stable and efficient reporter systems. Ultimately, the most appropriate approach should be determined by the intended application, with short-term HTS favoring strong signal intensity. In contrast, long-term *in vivo* studies demand constructs with minimal fitness cost and maximal stability.

## 4 Application of reporter-expressing viruses for antiviral drug screening

Traditional antiviral screening methods, such as cytopathic effect (CPE) assays and viral antigen-based techniques, are limited by low throughput and scalability. CPE assays rely on visual or spectrophotometric detection of virus-induced cell death and require several days of incubation and manual evaluation (Rasmussen et al., 2011; Jochmans et al., 2012). Similarly, ELISA and immunofluorescence-based antigen detection are time-consuming and labor-intensive (Grandien et al., 1985). These challenges are compounded by strict biosafety requirements for handling live viruses. Many high-priority pathogens, such as SARS-CoV-2, HIV, and influenza viruses, are classified as BSL-3 agents, necessitating specialized infrastructure and extensive safety training (Artika and Ma’roef, 2017; Kaufer et al., 2020; Wu et al., 2024).

Reporter-expressing viruses provide an efficient alternative, enabling real-time monitoring of the full viral life cycle with improved throughput and sensitivity (Manicassamy et al., 2010; Kato and Hishiki, 2016; Zhang et al., 2020; Fénéant et al., 2022). These systems have been instrumental in discovering antiviral compounds across various viral families. For example, a recombinant Ebola virus (EBOV) expressing eGFP allowed rapid high-throughput compound screening at BSL-2 containment (Towner et al., 2005; Ebihara et al., 2007; Hoenen et al., 2013).

Reporter viruses have also facilitated functional genomics approaches, including RNA interference (RNAi) and interferon-stimulated gene (ISG) screening. A FLuc-expressing classical swine fever virus (CSFV) was used to identify antiviral siRNAs efficiently (Shen et al., 2014), while a luciferase-expressing EBOV improved siRNA screening sensitivity relative to wild-type virus (Martin et al., 2018). Reporter CSFV has also been applied to profile ISGs with antiviral activity (Wang et al., 2016).

Reporter viruses have been adapted for screening in 384- or 1536-well format. HIV-1 luciferase reporters enabled the identification of integrase inhibitors such as dolutegravir by allowing real-time quantification of replication (Blair et al., 2005; Puig-Basagoiti et al., 2005). Luciferase-expressing hepatitis C virus (HCV) replicons accelerated the development of direct-acting antivirals (DAAs) such as sofosbuvir, targeting the NS5B RNA-dependent RNA polymerase (Koutsoudakis et al., 2006; Jones et al., 2007; Sofia et al., 2012; Chan et al., 2013; Hu et al., 2014; Tabata et al., 2020). A NanoLuc-expressing influenza A virus facilitated the screening of over 142,000 compounds, identifying novel influenza inhibitors (Weisshaar et al., 2016). Similarly, luciferase-expressing replicons were key to identifying remdesivir’s activity against SARS-CoV-2 (Eastman et al., 2020) and revealed mycophenolic acid as a Zika virus inhibitor (Pereira et al., 2023).

Primary antiviral screening campaigns often leverage compound libraries such as synthetic chemical collections (e.g., ChemDiv, Enamine REAL Space), FDA-approved drug libraries (e.g., Prestwick), or natural product repositories (e.g., NCI Natural Products Repository). Reporter viruses facilitate rapid screening of these large libraries by providing robust, quantitative readouts that reduce biosafety burdens and improve assay reproducibility.

When designing reporter-based HTS assays, the choice between fluorescent and bioluminescent reporters requires balancing sensitivity, cost, and assay logistics. FPs (e.g., eGFP, mCherry) are substrate-independent and inexpensive, enabling continuous live-cell imaging and multiplexing. However, their signal-to-background ratio is often lower, and they are less sensitive than luciferase-based reporters. In contrast, NanoLuc provides orders-of-magnitude higher sensitivity and superior dynamic range, making it particularly well suited for detecting low levels of viral replication (Hall et al., 2012). The trade-off is the requirement for exogenous substrate addition (e.g., furimazine), which increases assay cost and introduces additional liquid handling steps in large-scale campaigns.

Several experimental parameters strongly influence assay robustness. For fluorescent assays, using phenol-red-free media such as FluoroBrite significantly decreases background autofluorescence, thereby enhancing signal detection. Plate reader optics, well geometry, and incubation timing can further impact assay performance (Theodossiou et al., 2024). For luciferase-based assays, signal stability differs by enzyme: FLuc produces flash-type kinetics, whereas NanoLuc and engineered variants provide sustained glow-type kinetics. These properties must be considered when optimizing assay windows, minimizing false positives, and ensuring reproducibility in high-density plate formats (384- and 1536-well).

Beyond *in vitro* drug discovery, reporter viruses are invaluable for *in vivo* evaluation of antiviral efficacy. FPs and luciferases enable longitudinal monitoring of viral spread and therapeutic response without the need for terminal sampling. However, standard green and yellow FPs suffer from limited tissue penetration and high autofluorescence, reducing sensitivity in deep tissues. Far-red and near-infrared FPs (e.g., mKate2, iRFP, mNeptune) address these limitations by providing superior penetration and lower background (Shcherbo et al., 2007, 2009; Shcherbakova and Verkhusha, 2013). Bioluminescent reporters such as NanoLuc further enhance *in vivo* sensitivity due to their high signal-to-background ratios. Additionally, tissue-clearing techniques such as CLARITY and iDISCO allow three-dimensional mapping of infection and therapeutic outcomes in whole organs (Eliat et al., 2022; Frenkel et al., 2023; Richardson and Lichtman, 2015). While these methods expand the scope of reporter viruses, careful selection of reporters and imaging modalities remains crucial for translational applications.

In summary, reporter-expressing viruses have become central tools for antiviral drug discovery by bridging phenotypic HTS with functional genomics, drug repurposing, and translational *in vivo* studies. Their ability to deliver rapid, quantitative, and scalable readouts while also highlighting important practical considerations underscores their versatility in the development of next-generation antivirals. **Table 4** summarizes key studies utilizing reporter viruses for antiviral discovery.

**Table 4:** Application of reporter-expressing viruses for screening of antiviral compounds.

Reporter molecule	Target	Insertion position	Source for Screening	Inhibitor	Reference
ZsGreen1	Crimean-Congo hemorrhagic fever virus	NP N terminal (S genome segment)	Compound library (38 nucleoside analogs)	2’-deoxy-2’-fluorocytidine	(Welch et al., 2017)
ZsGreen1	Lujo Virus	N protein N-terminal (S genome segment)	Compound library (83 arenavirus inhibitors, broad-spectrum antivirals, FDA-approved library of compounds)	2’-deoxy-2’-fluorocytidine, apilimod, AVN-944, brequinar, BX-795, favipiravir	(Welch et al., 2021)
ZsGreen1	Sosuga virus	M N-terminal	Compound library (40 compounds including nucleoside analogs and viral replication inhibitors)	2’-deoxy-2’-fluorocytidine, 6-azauridine	(Welch et al., 2018)
ZsGreen1	Lassa virus	Between NP-GPC (S segment)	The DIVERSet-CL and macrocycle compound libraries (ChemBridge, San Diego, CA) contained 50,000 and 9676 compounds)	Macrocycle 12895623 (Mac128)	(Aida-Ficken et al., 2024)
ZsGreen	Lassa virus, Ebola virus	VP40 N-terminal	Nucleoside analogs (Sigma-Aldrich, Selleckchem, U.S. Pharmacopeia, Carbosynth, and Emory Institute of Drug Development (EIDD), 27 compounds)	6-azaurdine, 2’-deoxy-2’-fluorocytidine	(Welch et al., 2016)
eGFP	Enterovirus 71	5’UTR-VP4 (N-terminal)	Natural compound library (Weikeqi Biotech, China, 1,042 compounds)	Fangchinoline	(Zhang et al., 2024)
eGFP	Ebinur lake virus	5’UTR	Natural compound library (Weikeqi Biotech, China, 96 compounds)	Clinodiside A, secoxyloganin, disogluside	(Ren et al., 2022)
eGFP	Japanese encephalitis virus	C N-terminal	FDA-approved drug library (1,443 compounds)	Lonafarnib, cetylpyridinium chloride, cetrimonium bromide, nitroxoline hexachlorophene	(Zhang et al., 2020)
eGFP, NanoLuc	Parainfluenza virus 5	Between V/P and M	Common drug compounds (5 compounds)	Ribavirin	(Liu et al., 2021b)
eGFP	Enterovirus 71, coxsackievirus 16 (Picornaviridae)	Between 5’-UTR – VP4	Natural product library (National Institutes for food and drug control, Beijing, China, 400 compounds)	Luteolin, galangin, quercetin	(Xu et al., 2014)
GFP	Influenza A	Inserted into NA	Natural product compound (from traditional Mongolian medicine, 18 natural compounds)	Kaempferide, Curcumin, Cardamonin	(Nie et al., 2024)
GFP	Zika virus	C C-terminal	Plant sourced compound library (TargetMol, L4600, 974 compounds)	Homoharringtonine, bruceine D, dihydroartemisinin, digitonin	(Zhang et al., 2021)
mNeonGreen	Influenza D	Between NS1 and NS2	Compound library (COVID box, 80 compounds)	Ruxolitinib	(Probst et al., 2023)
FLuc	Hepatitis C	5’UTR	Compound library (>40 000 novel-structure compounds)	IB-32	(Niu et al., 2015)
FLuc	Murine coronavirus	Between E and M	Compound library (NIH Clinical Collection, 727 compounds)	Homoharringtonine, hexachlorophene	(Cao et al., 2015)
FLuc, RLuc, GLuc, NanoLuc (Splitted)	Hepatitis B	N-terminal (plus G_4_S92 linker)	Compound library (672 compounds)	Arbidol, 20-deoxyngenol	(Wei et al., 2018)
FLuc, NanoLuc	Influenza A, Respiratory Syncytial Virus	IAV: NanoLuc inserted behind PB2 RSV: FLuc+SMASh tag inserted before NS1	Compound library (LOPAC library, 1280 compounds)	Ribavirin, zanamivir, fenretinide, BNTX-7 (opioid receptor antagonist)	(Yan et al., 2015)
RLuc	Chimeric Hepatitis C	Between NS5A – NS5B	Compound library (1,175,504 compounds)	(17 inhibitors)	(Wichroski et al., 2012)
RLuc	Semliki Forest virus	Between nsP3 and nsP4	29 nucleoside analogs	3’-azido-3’-deoxyadenosine, 3’-amino-3’-deoxyadenosine	(Pohjala et al., 2008)
GLuc	Hepatitis E	Replacing ORF2	Compound library (FDA-approved compounds, 767 compounds)	Ciprofloxacin	(Nishiyama et al., 2019)
NanoLuc	SARS-CoV-2	Replacing ORF7	Compound library (44 compounds including nucleoside analogs, antivirals, and other classes of drugs)	Nelfinavir, rupintrivir, cobicistat	(Xie et al., 2020)
NanoLuc	Dengue virus	5’-UTR	Compound library (12,000 compounds)	BP34610	(Yang et al., 2019)
NanoLuc	Influenza A	PA C-terminal	Compound library (ChemBridge [100,000 compounds], ChemDiv [30,000 compounds], Emory Institute for Drug Development (EIDD) [1,155 compounds], Kansas University [11,520 compounds])	GRP-71271, GRP-103594, GRP-115249	(Weisshaar et al., 2016)
NanoLuc	JEV	Inserted before C	Antiviral drug library (Selleck, 227 compounds)	Cepharanthine	(Yin et al., 2024)
NanoKAZ	HEV	Inserted into HVR of ORF1	Compound library (FDA-approved drugs, 765 compounds)	Gefitinib, chlorpromazine, azithromycin, ritonavir	(Primadharsini et al., 2022)
Katushka	Rift Valley fever virus	Replacing NSs ORF	Compound library (Chemical Biology Consortium Sweden (CBCS), 28,437 compounds)	3-(2,3-Diphenyl-1H-indol-1-yl)-N′-(3-hydroxybenzylidene)propanohydrazide, 1-(3,5-Di-tert-butyl-4-hydroxyphenyl)-2-(2-imino-3-(4-methylbenzyl)-2,3-dihydro-1H-benzimidazol-1-yl)ethanone hydrobromide, 4-Hydroxy-3-methoxybenzaldehyde (4-(3,4-dimethoxyphenyl)-6-phenyl-2-pyrimidinyl)hydrazone, 1-(3,4-Dichlorophenyl)-3-(4-(3,4-dimethoxyphenyl)-1-methylpyrrolidin-3-yl)urea, 2-Chloro-N-(2-(5-(trifluoromethyl)-1,3,4-thiadiazol-2-ylcarbamoyl)phenyl)benzamide, 2-(4-Bromo-5-chlorothiophene-2-sulfonamido)-N-(4-fluorophenyl)benzamide	(Islam et al., 2016)
mRFP1	Human adenovirus	Replacing E3	Compound library (Prestwick Chemical Library, Prestwick chemical, 1200 FDA-approved compounds)	Digoxin, digitoxigenin, lanatoside C, cytarabine, dexamethasone acetate, flunisolide	(Saha et al., 2019)
HiBiT	Influenza A	NEP N-terminal	58 pre-purified TCM extracts	*Trametes robiniophila* (50% methanol fraction), *Ganoderma* (water fraction), wild *Phellinus igniarius* (ethyl acetate fraction),	(Zhang et al., 2023a)
HiBiT	Yellow fever virus	NS-1 N terminal	Anti-flavivirus compounds (5 compounds)	7-deaza-2’-C-methyladenosine, celgosivir, nelfinavir	(Sanchez-Velazquez et al., 2020)

## 5 Challenges in the application of reporter-expressing viruses for drug screening

While reporter-expressing viruses have become powerful tools for antiviral drug discovery, their effective use in HTS is not without challenges. For such assays to be reproducible and translatable, issues related to biosafety, genetic stability, expression strategy, and detection methods must be addressed. Below, we highlight key limitations with direct implications for antiviral drug screening and HTS workflows.

### 5.1 Handling of recombinant viruses

In HTS campaign involving thousands of compounds, biosafety and scalability are major concerns. Handling infectious reporter-expressing viruses requires specialized containment facilities (BSL-3/4), necessitating trained personnel and strict biosafety protocols (Kaufer et al., 2020; Wu et al., 2024). These requirements constrain large-scale screening, particularly in resource-limited environments. Moreover, the use of live viruses inherently poses risks of unintended exposure, and recombinant viruses may display instability or reduced infectivity compared to wild-type strains, resulting in variability in assay performance (Rasmussen et al., 2011).

To address these challenges, several biosafety-compatible alternatives have been developed. One approach involves creating single-cycle or replication-defective reporter viruses by replacing virulence-related genes with reporter genes. These constructs can often be handled under BSL-2 conditions while still mimicking many aspects of viral replication (Ge et al., 2008; Iro et al., 2009; Todt et al., 2020). For example, Huh7 cells expressing mCherry-tagged IPS-1 were used to identify anti-HCV compounds from natural products (Zheng et al., 2021). Similarly, LASV vRNP-ZsGreen fusion constructs facilitated screening of FDA-approved drugs, leading to the discovery of afatinib (Mizuma et al., 2022).

Another approach is the use of replicons, subgenomic viral RNAs lacking structural protein genes. They offer another safe and versatile platform for dissecting specific stages of viral life cycle (replication, translation, or transcription) and are particularly valuable for HTS. For instance, they are used to identify replication inhibitors, whereas VLP-based systems enable screening for entry inhibitors. Replicons have been widely applied for HCV (Lee et al., 2004; Hao et al., 2007), IAV (Li et al., 2017), WNV (Zhang et al., 2017a), DENV (Hsu et al., 2012; Leardkamolkarn and Sirigulpanit, 2012; Kato et al., 2014, 2019; Yang et al., 2014), YFV (Patkar et al., 2009), CHIKV (Pohjala et al., 2011), HEV (Netzler et al., 2019; Nishiyama et al., 2019; Qu et al., 2019; Li et al., 2020b; Nimgaonkar et al., 2021), ZIKV (Li et al., 2018), SARS-CoV-2 (He et al., 2021), poliovirus (Ford Siltz et al., 2014), and rotavirus (Kanai et al., 2019). Together, these surrogate systems provide practical routes to large-scale phenotypic screening without requiring maximum biosafety containment.

Host cell-based reporter systems also enable efficient screening under lower biosafety requirements. For example, a stable NF-κB luciferase-expressing cell line was used to screen over 101,000 compounds, leading to the discovery of E567, a potential anti-LCMV and anti-HSV-1 compound (Zhou et al., 2010). Other examples include reporter cells used to screen anti-ASFV agents (Zhang et al., 2023b) and MERS-CoV RdRp inhibitors such as remdesivir (Min et al., 2020). Similarly, a HeLa cell line expressing FLuc under IAV UTR control was used to identify HA-mediated fusion inhibitors (Zhang et al., 2012).

Finally, virus minigenome and transcription/replication-competent virus-like particle (trVLP) systems, such as those used for LASV, allow modeling of viral replication in a noninfectious context suitable for drug screening (Qing et al., 2010; Watt et al., 2014; Welch et al., 2016). These platforms combine safety with biological relevance, providing practical alternatives for large-scale antiviral screening without requiring high-level containment.

### 5.2 Genetic instability

Genetic instability remains a persistent challenge in reporter virus construction. Inserting large reporter genes, such as GFP, can interfere with viral genome packaging or protein processing, resulting in decreased replication or reporter loss (Manicassamy et al., 2010). For example, IAV expressing GFP in the NS segment exhibited attenuation and lost fluorescence over serial passages. Instability can also arise at the molecular cloning stage, particularly in flaviviruses and retroviruses, where reporter cassettes are prone to deletion or rearrangement during bacterial propagation of plasmid clones (Aubry et al., 2015).

To mitigate these issues, smaller and brighter reporters are increasingly favored. NanoLuc (~19 kDa), picALuc (~13 kDa), and mini-FAPs (~14 kDa) impose a reduced genetic burden compared to GFP (~27 kDa), thereby improving the likelihood of stable propagation (Heaton et al., 2013; England et al., 2016; Xie et al., 2020; Fang et al., 2021, 2022; Liu et al., 2021b). Complementation tags such as HiBiT (11 amino acids) allow an even more compact option, enabling sensitive detection with minimal genomic disruption (Nagashima et al., 2023; Zhang et al., 2023b).

In addition, self-cleaving 2A peptides such as PTV-1 help maintain viral protein function while co-expressing reporters (Reuther et al., 2015). Adaptive passaging *in vivo* has restored viral replication while preserving reporter stability (Caì et al., 2018). Finally, deep insertional mutagenesis and genome-wide scanning approaches have been used to systematically map regions that tolerate foreign sequence insertions without impairing replication (Bakhache et al., 2024), providing a rational framework for engineering genetically stable reporter-expressing viruses.

### 5.3 Expression strategy

Careful selection of insertion sites and expression strategies is crucial for minimizing attenuation. Random insertion screening has been used to identify viable reporter locations in HCV (Moradpour et al., 2004) and HEV (Oechslin et al., 2023). In HEV, only the hypervariable region tolerated insertions, whereas in HCV, NS5A allowed stable integration.

Common strategies include fusion of reporter to viral proteins, additional transcription unit (ATU)-based expression, and IRES-driven bicistronic expression (Falzarano et al., 2014). To overcome co-translation challenges, reporters have been fused to ER-anchored proteins with nuclear localization signals that are cleaved during infection by viral proteases (Medin et al., 2015; Hsieh et al., 2017; McFadden et al., 2018). Other systems replace viral protein substrates with protease-specific cleavage motifs to enable infection-dependent reporter activation (Pahmeier et al., 2021).

Conditional systems such as Cre-*lox*P, enable reporter expression only in cells infected by a Cre-expressing virus and have been used for anti-HCV screening (Hu et al., 2014). Pseudovirus-based RNA delivery approaches have also been employed to screen for viral entry inhibitors (Xu et al., 2022).

### 5.4 Signal attenuation and reporter interference

A major challenge in applying reporter-expressing viruses is the attenuation or distortion of reporter signals, particularly during *in vivo* imaging and HTS. Several technical and biological factors can compromise detection and reduce assay sensitivity.

Fluorescent reporters such as GFP, while compact and non-toxic (Agungpriyono et al., 1999), suffer from poor performance in deep tissue imaging because visible light is strongly absorbed and scattered by biological tissues (Deliolanis et al., 2008). Autofluorescence from endogenous molecules or pigment-rich natural products (e.g., chlorophyll, polyphenols) further increase background noise and decrease signal clarity (Croce, 2021). These limitations restrict the application of GFP-like proteins largely to cell culture systems and *ex vivo* assays (Tran et al., 2013). Recent advances in far-red and near-infrared FPs (e.g., miRFP670) offer improved penetration and reduced background autofluorescence (Shcherbakova and Verkhusha, 2013), although their performance in viral systems is still being optimized.

Bioluminescent reporters address many of these limitations, as they do not require external excitation and thus exhibit higher sensitivity and deeper tissue penetration. FLuc with D-luciferin remains widely used in small animal models (Barry et al., 2012), while NanoLuc provide stronger intensity with a smaller genetic footprint, suited for compact viral genomes (Hall et al., 2012). Red-shifted and dual-color luciferases further expand applications by enabling multiplex imaging and better performance in tissue environments (Caysa et al., 2009; Mezzanotte et al., 2011).

For HTS, additional experimental considerations are necessary to minimize background interference. The use of phenol-red-free media such as FluoroBrite significantly reduces background fluorescence and improve plate-based assay performance (Ettinger and Wittmann, 2014). Ensuring substrate stability and uniform delivery is also critical for luciferase-based assays, where the need for exogenous substrates (e.g., D-luciferin, furimazine) increases cost but yields higher sensitivity than fluorescent systems.

Emerging approaches such as tissue-clearing and spectral unmixing algorithms may expand fluorescent reporter application *in vivo.* Rational engineering for optimization of luciferases and FPs to enhance brightness, spectral range, and stability (Chu et al., 2025) will help overcome current signal attenuation challenges and broaden the applicability of reporter-expressing viruses in both mechanistic studies and drug discovery.

### 5.5 Data analysis and hit validation

HTS using reporter-expressing viruses produces complex datasets that demand robust computational workflow for reliable hit identification. Automated imaging assays using fluorescent or luminescent reporters rely on multiparametric feature extraction, capturing not only intensity but also cell morphology, nuclear changes, cytopathic effects, and spatial signal distribution. This approach, known as High-Content Analysis (HCA), provides a richer phenotypic profile than simple bulk intensity measurements, thereby enhancing sensitivity in detecting subtle antiviral effects (Taylor and Giuliano, 2005).

Open-source platforms such as CellProfiler and ImageJ/Fiji, along with commercial platforms like Columbus, MetaXpress, IN Cell Developer, are widely used for viral HCA. These tools perform normalization, background correction, cell segmentation, and quantification of infection dynamics across thousands of wells. Integration of HCA pipelines with statistical quality control metrics such as the *Z’*-factor ensures assay robustness and reproducibility (Pearson et al., 2022). HCA’s ability to capture diverse cellular features including reporter intensity, cell viability, and morphological changes makes it particularly powerful for distinguishing genuine antiviral effects from cytotoxic artifacts.

Although reporter-expressing viruses model the complete infection cycle and are therefore suited for identifying broad-spectrum inhibitors, they may overlook compounds targeting specific replication stages. Therefore, candidate hits from HTS must undergo orthogonal validation, including wild-type virus infection assays, minigenome systems, or stage-specific replication assays, to confirm mechanism and efficacy (Edwards et al., 2015; Fu et al., 2019; Todt et al., 2020) (Magden et al., 2005; García et al., 2017; Rampersad and Tennant, 2018).

In summary, coupling reporter-expressing viruses with advanced HCA pipelines has redefined phenotypic antiviral discovery, enabling scalable, quantitative, and mechanistically informed screening. Future analytical directions include the adoption of machine learning–assisted image analysis and deep phenotypic profiling, which promise enhanced hit prioritization and mechanistic insight while maintaining assay scalability and reproducibility.

## 6 Future development

Reporter-expressing viruses are poised to further revolutionize virology, antiviral drug discovery, and therapeutic development by enabling real-time monitoring and quantification of viral replication, pathogenesis, and host-pathogen interactions in both *in vitro* and *in vivo* systems (Jones et al., 2010; Manicassamy et al., 2010; Tran et al., 2013; Kato and Hishiki, 2016). Several key areas hold promise for further development and expansion of reporter virus applications.

A central focus for future advancement will be the development of reporter proteins with improved sensitivity and specificity. Enhancing the signal-to-noise ratio and minimizing background interference are critical objectives. These objectives include engineering smaller reporter genes with brighter emissions, greater photostability, and minimal cytotoxicity. New FPs and BPs such as NanoLuc and mNeonGreen offer excellent characteristics in this regard, providing more durable signals and superior optical performance for longitudinal studies (Tamura et al., 2018; Deng et al., 2021; Mathieu et al., 2021; Fénéant et al., 2022; Liu et al., 2023; Bakhache et al., 2025).

Dual-reporter and multiplex systems represent another frontier. These technologies can simultaneously track multiple biological parameters such as distinct viral replication events, co-infections, or virus-host interactions. For example, one reporter may indicate viral entry while another tracks replication or immune signaling, offering a more comprehensive understanding of infection dynamics (Wu et al., 2011).

Additionally, expanding the application of reporter systems to underrepresented viral families, such as arboviruses or emerging coronaviruses, remains a significant opportunity. Current systems are often virus-specific, with extensive development for influenza, HIV, or HCV. Broadening the technology’s applicability to zoonotic and neglected viruses will enhance preparedness for future outbreaks.

The integration of reporter-expressing viruses with automated HTS platforms is expected to grow. Robotics and AI-based systems will facilitate the rapid evaluation of thousands of compounds, streamlining the drug discovery process (Lee et al., 2013; Zhang et al., 2020; Sacramento et al., 2025). AI-driven image analysis, deep mutational scanning, and next-generation high-content imaging will enhance the reliability and mechanistic depth of reporter virus–based HTS in antiviral drug discovery. The demand for scalable and rapid antiviral screening has accelerated the adoption of such technologies.

Enhanced imaging compatibility will also benefit *in vivo* applications. Reporter viruses optimized for noninvasive systems such as bioluminescence imaging or advanced fluorescence imaging will improve the ability to observe viral dissemination and tropism in real time within living organisms. For instance, bioluminescent IAV expressing NanoLuc has been used to monitor infection progression and tissue specificity in murine models, offering critical insight into host-pathogen dynamics (Manicassamy et al., 2010; Tran et al., 2013; Smith et al., 2025).

The use of reporter viruses may transform personalized medicine far beyond basic and translational research. The genome-specific reporter viruses could be used to infect organoid cultures or patient-derived cells for individualized antiviral testing. Using host factors, viral genotype, and predicted drug response provides physicians the ability to personalize treatments for each individual. HCV replicons expressing luciferase have already been employed with primary human hepatocytes to establish the best treatment regimens based on the viral profiles of each patient (Koutsoudakis et al., 2006; Steinmann et al., 2007; Tabata et al., 2020).

The future of reporter-expressing viruses largely depends on ongoing inventions to enhance sensitivity, adaptability, and applicability. As these technologies develop, they will progressively guide real-time antiviral strategies, host-pathogen modeling, and precision virology.

## 7 Conclusion

Reporter-expressing viruses have revolutionized antiviral drug discovery by allowing real-time, high-throughput, and quantitative assessment of viral replication, host-pathogen interactions, and drug efficacy. By integrating fluorescent and bioluminescent reporters into viral genomes, these systems offer sensitive, rapid, and scalable platforms for screening compound libraries and validating antiviral targets both *in vitro* and *in vivo*.

Despite these advantages, several challenges persist, including maintaining genetic instability, minimizing viral attenuation, ensuring biosafety, and reducing signal interference. Careful optimization of reporter selection, insertion sites, and expression strategies is essential., while complementary systems, such as replication-defective systems, reporter-expressing cells, viral replicons, and minigenomes, further enhance safety and flexibility for antiviral testing under lower containment levels.

Future development will focus on improving signal strength and specificity, reducing cytotoxicity, and enhancing imaging compatibility for *in vivo* imaging. The emergence of compact, bright, and stable reporters (e.g., NanoLuc, mNeonGreen) and multiplexed dual-reporter designs will enable simultaneous measurement of multiple viral and cellular events. Integration with patient-derived organoids and primary cells may also open avenues for individualized antiviral screening and precision medicine.

In summary, reporter-expressing viruses represent a pivotal technology leap in antiviral research. Continued innovation in reporter design, automation, and imaging will sustain their impact as indispensable tools for precision virology and the development of next-generation antiviral therapeutics.
